# Cyclin K regulates prereplicative complex assembly to promote mammalian cell proliferation

**DOI:** 10.1038/s41467-018-04258-w

**Published:** 2018-05-14

**Authors:** Tingjun Lei, Peixuan Zhang, Xudong Zhang, Xue Xiao, Jingli Zhang, Tong Qiu, Qian Dai, Yujun Zhang, Ling Min, Qian Li, Rutie Yin, Ping Ding, Ni Li, Yi Qu, Dezhi Mu, Jun Qin, Xiaofeng Zhu, Zhi-Xiong Xiao, Qintong Li

**Affiliations:** 1 Department of Obstetrics and Gynecology, West China Second University Hospital, Key Laboratory of Birth Defects and Related Diseases of Women and Children, Ministry of Education, College of Life Sciences, State Key Laboratory of Biotherapy and Collaborative Innovation Center of Biotherapy, Sichuan University, 610041 Chengdu, China; 2 Department of Obstetrics and Gynecology, West China Second University Hospital, Sichuan University, 610041 Chengdu, China; 3 Department of Pathology, Hospital 363 of Aviation Industry Corporation of China, 610041 Chengdu, China; 4 Department of Pediatrics, West China Second University Hospital, Sichuan University, 610041 Chengdu, China; 5 Center of Growth, Metabolism and Aging, Key Laboratory of Bio-Resource and Eco-Environment, Ministry of Education, College of Life Sciences, Sichuan University, 610064 Chengdu, China; 6 Sichuan Cunde Therapeutics, 610093 Chengdu, China; 7 Key Laboratory of Stem Cell Biology, Chinese Academy of Sciences Center for Excellence in Molecular Cell Science, Institute of Health Sciences, Shanghai Institutes for Biological Sciences, University of Chinese Academy of Sciences, 200031 Shanghai, China

## Abstract

The assembly of prereplicative complex (pre-RC) during G1 phase must be tightly controlled to sustain cell proliferation and maintain genomic stability. Mechanisms to prevent pre-RC formation in G2/M and S phases are well appreciated, whereas how cells ensure efficient pre-RC assembly during G1 is less clear. Here we report that cyclin K regulates pre-RC formation. We find that cyclin K expression positively correlates with cell proliferation, and knockdown of cyclin K or its cognate kinase CDK12 prevents the assembly of pre-RC in G1 phase. Mechanistically we uncover that cyclin K promotes pre-RC assembly by restricting cyclin E1 activity in G1. We identify a cyclin K-dependent, novel phosphorylation site in cyclin E1 that disrupts its interaction with CDK2. Importantly, this antagonistic relationship is largely recapitulated in cyclin E1-overexpressing tumors. We discuss the implications of our findings in light of recent reports linking cyclin K and CDK12 to human tumorigenesis.

## Abstract (web summary)

Prereplicative complex (pre-RC) formation during G1 is fundamental for cell replication. Here the authors report a role for cyclin K in regulating pre-RC formation in mammalian cells by affecting cyclin E1 activity.

## Introduction

Precise regulation of DNA replication is paramount to sustain cell proliferation and maintain genomic integrity^1^. The prereplicative complex (pre-RC) is assembled only in G1 phase, a process referred as replication origin licensing, to ensure single round of DNA replication per cell cycle^2^. The pre-RC is formed by sequential recruitment of the origin recognition complex (ORC1–ORC6), cell division cycle 6 (CDC6), chromatin licensing and DNA replication factor 1 (CDT1), and finally the helicase complex comprising minichromosome maintenance proteins (MCM2–MCM7)^3^. Mechanisms to prevent licensing in G2/M and S phases are well appreciated. Under physiological conditions, mitotic kinase activities stabilize CDT1 in G2/M phase by promoting its interaction with Geminin protein (encoded by *GMNN*). Meanwhile, Geminin restricts licensing by preventing CDT1 from loading onto DNA, as well as interacting with MCM2–MCM7^4,5^. During DNA synthesis, CDT1 is rapidly degraded on DNA in a PCNA-dependent manner to ensure no new pre-RCs can be assembled in S phase^6^. CDC6 and the origin recognition complex may also be regulated by various mechanisms to further prevent untimely assembly of pre-RCs^3^. In contrast, how cells ensure efficient pre-RC assembly during G1 phase is less clear^7^.

Oncogenic signals can sabotage the precise regulation of pre-RC assembly^2^. Early studies have established that cyclin E1 overexpression is detrimental to pre-RC formation in G1 phase even in highly transformed cancer cells^8^. This is believed to lead to insufficient licensing, subsequent genetic instability, and eventually tumorigenesis^9–12^. Recent whole-genome sequencing studies strengthened this notion by demonstrating that cyclin E1 is amplified in a substantial portion of human high-grade serous ovarian cancer^13,14^, a disease characterized by genomic instability^15^. On the other hand, cyclin E1 is required for efficient pre-RC assembly when cells exit from G0 phase (quiescence) to enter the cell cycle^16,17^. These functions of cyclin E1 are partly independent of its cognate kinase CDK2, although the exact molecular mechanisms remain unknown^16,17^. Taken together, these observations demonstrate that the activity of cyclin E1 needs to be tightly regulated during pre-RC assembly in G1.

Human *cyclin K* was originally cloned by its ability to restore viability in yeast cells lacking all G1-cyclin proteins^18^. Its function nevertheless remains poorly understood. A recent study established that genetic ablation of *cyclin K* results in lethality at very early stage of mouse embryogenesis^19^. Subsequent studies suggested that cyclin K may regulate transcription of several genes^19–21^. However, it is not clear whether the transcriptional defect is a direct effect, nor can it explain the early embryonic lethal phenotype in mice. We previously found that cyclin K protein is hardly detectable in nonproliferative human and murine adult tissues^22^, whereas it is highly expressed in fast growing stem cells^23^. These observations prompted us to investigate the role of cyclin K in cell proliferation. Here we report that cyclin K regulates pre-RC formation in mammalian cells by regulating cyclin E1 activity.

## Results

### Cyclin K expression positively correlates with proliferation

**Fig. 1:**
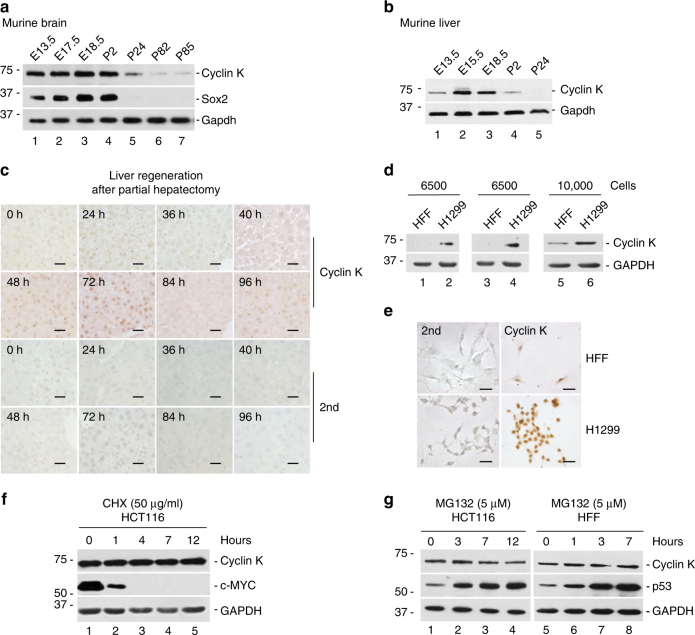
Cyclin K expression positively correlates with proliferation. **a** Analyses of cyclin K protein expression during murine brain development by immunoblotting. Cyclin K protein expression in embryonic (E) and postnatal (P) murine brains correlated with that of Sox2, a marker of neural progenitor cell proliferation. **b** Analyses of cyclin K protein expression by immunoblotting during murine liver development. **c** Cyclin K expression detected by immunochemistry during the process of murine liver regeneration in vivo. 2nd, immunochemistry using secondary antibodies alone. 0 h denotes samples collected immediately after partial hepatectomy. Scale bar, 40 μm. **d** Comparison of cyclin K by immunoblotting in normal and H1299 cancer cells using equal cell numbers as loading control. HFF, neonatal human foreskin fibroblast. **e** Cyclin K expression detected by immunochemistry in normal and H1299 cancer cells. HFF, neonatal human foreskin fibroblast. Scale bar, 40 μm. **f** Time course analyses of cyclin K expression by immunoblotting in HCT116 cells treated with protein synthesis inhibitor cycloheximide (CHX, 50 μg/ml). **g** Time course analyses of cyclin K expression by immunoblotting in cells treated with proteasome inhibitor MG132 (5 μM) in human normal and HCT116 cancer cells. HFF, neonatal human foreskin fibroblast. Experiments were repeated for three times (**a**–**c**), and more than three times when cell lines were used (**d**–**g**). Representative results are shown

Previously we have shown that in adult nonproliferative tissues, cyclin K expression is extremely low^22^. Here we extended the analysis to several biological contexts to establish the correlation between cyclin K expression and cell proliferation. During murine embryogenesis, neural progenitor cells divide rapidly, a process controlled by Sox2 transcription factor^24^. The expression pattern of cyclin K mimicked that of Sox2 during embryonic (E) and postnatal (P) stages (Fig. 1a). Developmentally regulated expression of cyclin K was also observed in murine liver (Fig. 1b). Growth of both organs significantly slows down postnatally, coinciding with diminished expression of cyclin K (Fig. 1a, b). Expression of cyclin K was further examined during the course of liver regeneration after partial hepatectomy^25^. In this classic in vivo model, hepatocytes enter the cell cycle in a relatively synchronized manner, and divide once or twice to fully restore liver size around one week^26^. Cyclin K expression was steadily increased, peaked at 72 h, and subsided afterwards (Fig. 1c). This kinetics is consistent with established hepatocyte cell cycle entry and progression after hepatectomy. In addition, cyclin K expression was easily detectable in various human cancer cell lines (Fig. 1d and Supplementary Fig. 1a). Its expression appeared to be higher in cancer cells than in normal human foreskin fibroblasts (HFF) (Fig. 1d). Expression seemed fairly homogenous among cells, suggesting cell cycle-independent regulation (Fig. 1e). Consistently cyclin K protein was more stable than classic cyclin proteins that regulate cell cycle (cyclins A, B, and D), and not subjected to proteasome regulation (Fig. 1f, g). Previously, we found that cyclin K expression was much higher in embryonic stem cells (doubling time ~10 h) than in slowly proliferating dermal stem cells (doubling time ~60 h)^23^. Cyclin K expression is also hardly detectable in adult nonproliferative murine and human tissues^22^. These results collectively demonstrate that cyclin K expression positively correlates with cell proliferation status under multiple biological contexts.

Uncontrolled cellular proliferation is a hallmark of cancer cells^27^. A previous study suggested that cyclin K expression was controlled by p53^28^, a master regulator of cell proliferation. Therefore, we decided to investigate p53-cyclin K relationship. We found that cyclin K expression was easily detectable in various human cancer cell lines regardless of their p53 status (wild-type *p53* in HFF and HCT116, inactivated *p53* in HeLa, and *p53*-null in H1299 and HCT116 *p53−/−*) (Fig. 1d and Supplementary Fig. 1a). These observations seemed to be at odds with the previous study^28^, in which the authors concluded that the basal level, as well as induced expression of cyclin K is dependent on p53. To resolve this discrepancy, we used the same cell line, MCF7, as in the previous study. Ultraviolet radiation (UV) induced a robust accumulation of p53, whereas cyclin K protein level remained constant (Supplementary Fig. 1b). UV treatment also failed to induce cyclin K expression in wild-type and isogenic *p53*-null HCT116 cell lines (Supplementary Fig. 1c). Of note, cyclin K expression was identical with or without p53 (Supplementary Fig. 1c). We considered the possibility that cyclin K protein level might be so high in those cancer cells that it could not be further increased by external signals. We ruled out this possibility because neither protein nor mRNA level was induced by UV in HFF (Supplementary Fig. 1d), in which cyclin K expression is normally low. In addition, inhibition of proteasome activity did not increase cyclin K protein level, even though p53 protein was massively stabilized (Fig. 1g). Taken together, these results demonstrate that cyclin K is highly expressed in cancer cells, but not under the control of p53 as previously suggested.

### Cyclin K is required for cell proliferation

**Fig. 2:**
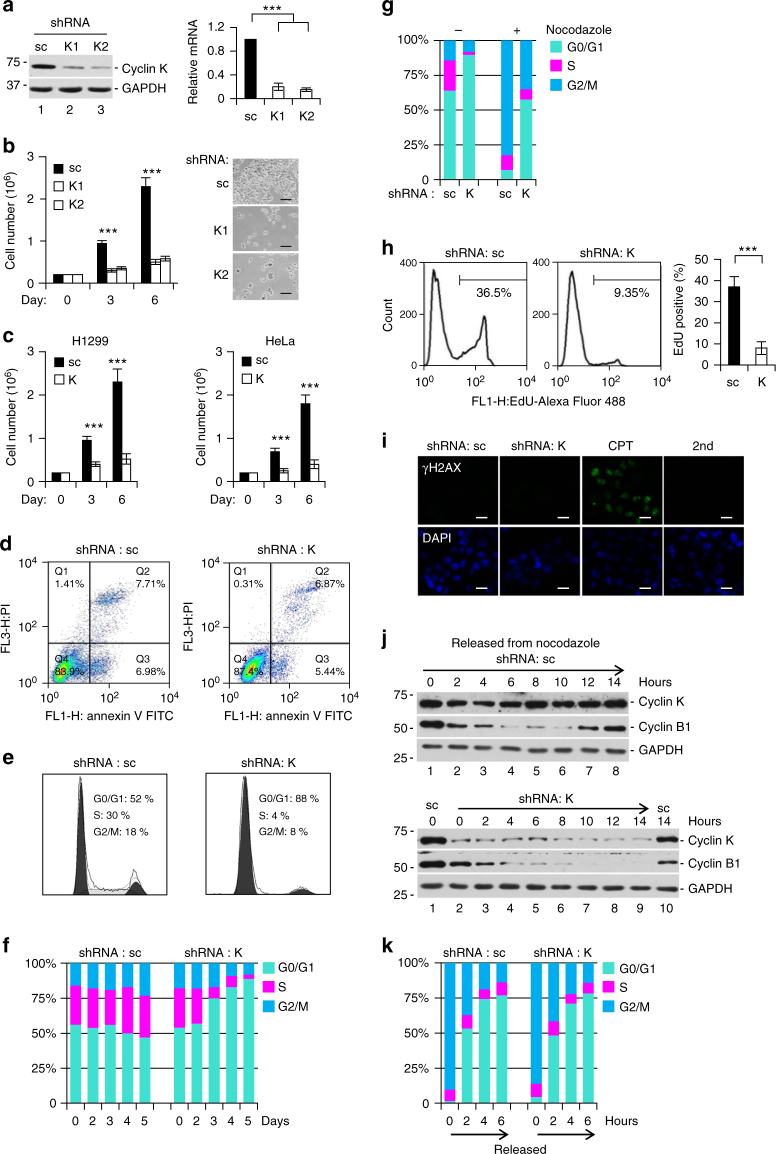
Cyclin K is required for cell proliferation. **a** Knockdown of cyclin K by two short-hairpin RNA constructs (shRNA, K1, K2) in HCT116 cells. sc, scrambled shRNA. Left panel, protein blot analysis. Right panel, qPCR analysis. Data are means ± SEM (*n* = 7) (***p < 0.001; Student’s *t*-test). **b** Time course analyses of cell number after cyclin K knockdown in HCT116 cells. Right panel, bright-field microscopy of cells at day 6. Scale bar, 200 μm. Data are means ± SEM (*n* = 5) (***p < 0.001; Student’s *t*-test). **c** Similar experiments as in **b** using H1299 and HeLa cells. Data are means ± SEM (*n* = 5) (***p < 0.001; Student’s *t*-test). **d** The percentage of Annexin V-positive cells (marker of early apoptosis) was similar with or without cyclin K knockdown in HCT116 cells. **e** The percentage of sub-G1 fraction (apoptotic cells) was similar with or without cyclin K knockdown in HCT116 cells. **f** Cell cycle profiling by fluorescence-activated cell sorting (FACS) analyses with or without cyclin K knockdown in HCT116 cells for up to 5 days. **g** Cell cycle profiling of HCT116 cells treated with or without cyclin K knockdown for 2.5 days, followed by 14-h nocodazole treatment (800 ng/ml). **h** Detection of EdU-positive HCT116 cells (proliferating cells) by FACS with or without cyclin K knockdown for 3 days. Quantitation was generated from five independent experiments. Data are means ± SEM (*n* = 6) (***p < 0.001; Student’s *t*-test). **i** Immunofluorescence analysis of γH2AX (marker of DNA damage) in HCT116 cells with or without cyclin K knockdown for 5 days. Camptothecin (CPT, 2 μM) was used as a positive control for DNA damage induction. Scale bar, 15 μm. **j** Time course analyses of cyclin B1 expression level in synchronized HCT116 cells with or without cyclin K knockdown for 2 days. Cells were synchronized at M phase (0 h), and then released into fresh medium. Lanes 2–9 in lower panel, cells with cyclin K knockdown. **k** Cell cycle profiling by FACS of HCT116 cells in **j**. All experiments were repeated at least three times and representative results are shown

The positive correlation between cyclin K expression and cell proliferative state promoted us to ask whether cyclin K might regulate cell proliferation. Two short hairpin RNA (shRNA) constructs were generated to knock down cyclin K expression (Fig. 2a). Both constructs were used in most experiments throughout this study, and generated similar results. For the simplicity of presentation, only results from one shRNA were shown for most experiments. Knockdown of cyclin K efficiently reduced total number of cells to a similar degree (Fig. 2b). Same results were seen in other cell lines, indicating a general phenomenon (Fig. 2c). The percentage of cells stained positively for annexin V (a marker of early apoptosis) was similar in control and knockdown cells, indicated that apoptosis was not the cause for reduced cell number (Fig. 2d). No obvious sub-G1 signal in FACS analysis further confirmed that knockdown did not trigger apoptosis (Fig. 2e). Robust G1 arrest was observed at day 3 following lentiviral delivery of shRNA (Fig. 2e). Time-course analysis showed that G1 arrest occurred between 2 and 3 days after shRNA delivery (Fig. 2f). A substantial portion of cells remained in G1, even after treatment with nocodazole at 2.5 days post shRNA delivery (Fig. 2g). These observations suggest that G1 arrest may be an immediate effect of cyclin K knockdown because it takes at least 1 day to express sufficient amount of shRNA to significantly reduce cyclin K mRNA, and cyclin K protein has a half-life longer than 12 h (Fig. 1f). Proliferation defect was further confirmed by significantly reduced DNA replication (measured by EdU incorporation) 3 days post shRNA delivery (Fig. 2h). Virtually no positive γH2AX signal was detected by immunofluorescence after 5 days (Fig. 2i), indicating that observed cell cycle arrest was unlikely caused by DNA damage. We and others have recently established that cyclin K interacts with CDK12 in cells^19,21,23,29^. Knockdown of CDK12 largely recapitulated effects caused by cyclin K knockdown (Supplementary Fig. 2), indicating that cyclin K functions with CDK12 to regulate cell proliferation. Taken together, our results demonstrate that cyclin K is required for cell proliferation.

To investigate immediate effects in a homogenous cell population, we developed a synchronization procedure in which nocodazole was added 2 days post shRNA delivery. Following experiments were carried out using this procedure unless otherwise specified. Generally, shRNA treatment in this time window efficiently reduced cyclin K protein level without triggering overt G1 arrest (Fig. 2j, k). Subsequent treatment with nocodazole usually arrested more than 95% of cells in G2/M phase. M-phase cells were then collected by panning, and released into fresh medium to initiate cell cycle progression. After release, cyclin B1 was degraded at normal kinetics (Fig. 2j), suggesting that cyclin K knockdown did not affect mitotic exit or G1 entry. This was also confirmed by FACS analysis (Fig. 2k).

### Cyclin K is loaded on chromatin in parallel with pre-RC

**Fig. 3:**
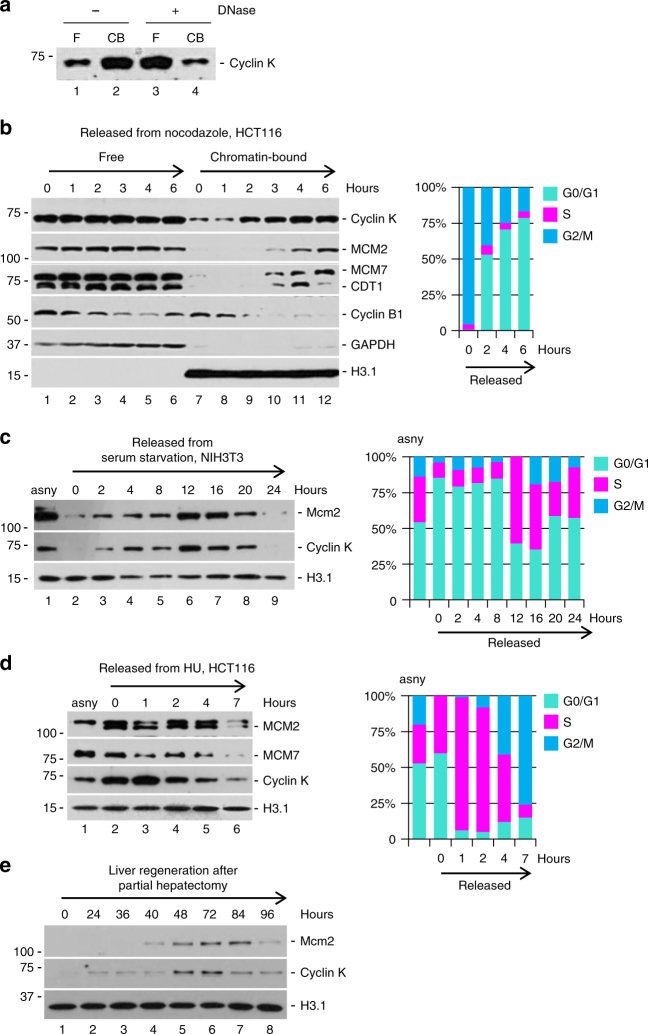
Cyclin K is loaded onto chromatin in parallel with pre-RC. **a** Cyclin K protein level detected by immunoblotting in soluble (free) and chromatin-bound (CB) fractions with or without DNase treatment. **b** Loading of cyclin K and components of prereplicative complex (CDT1, MCM2, and MCM7) onto chromatin. HCT116 cells were synchronized at M phase (0 h), and then released into fresh medium. Corresponding cell cycle profiling by FACS analyses was presented in right panel. **c** Loading of cyclin K and Mcm2 onto chromatin. NIH 3T3 cells were synchronized at G0 by serum starvation, and then released into fresh serum-containing medium. Corresponding cell cycle profiling by FACS analyses was presented in the right panel. **d** Loading of cyclin K, MCM2, and MCM7 onto chromatin. HCT116 cells were synchronized at G1/S boundary by hydroxyurea treatment (HU, 1 mM), and then released into fresh medium. Corresponding cell cycle profiling by FACS analyses was presented in right panel. **e** Loading of cyclin K and Mcm2 onto chromatin during the course of liver regeneration in vivo. 0 h denotes samples collected immediately after partial hepatectomy. All experiments were repeated more than three times and representative results are shown

Stepwise assembly of the prereplicative complex (pre-RC) is a critical cellular event in G1 phase. It commences at late M to early G1 phase, and completes before cells enter S phase^3^. Given the fact that cyclin K knockdown led to G1 arrest (Fig. 2), we investigated the relationship between cyclin K and pre-RC formation. Biochemical fractionation revealed that significant amount of cyclin K protein was present in the insoluble portion. Nuclease treatment released most cyclin K protein into supernatant (Fig. 3a), demonstrating that most cyclin K protein is associated with chromatin. We then asked whether loading of cyclin K onto chromatin correlated with pre-RC formation under various biological contexts. First, M phase-synchronized cells obtained by mitotic shake-off procedure were examined (Fig. 3b). FACS analysis demonstrated that more than 95% of cells were in M phase. These cells were then released into fresh medium to initiate cell cycle progression. Cells were collected at indicated time point. An aliquot of cells was used to isolate total cell lysate and the rest for FACS analysis. Total cell lysates at each time point were further separated into soluble and chromatin-bound fractions using previously established protocols^17,30^. Successful separation was demonstrated by GAPDH present only in soluble fractions and histone H3.1 solely in chromatin-bound fractions. As expected, cyclin B1 protein was rapidly degraded after release from nocodazole (indicating exit from M phase), and pre-RC assembly commenced at early G1 (indicated by loading of CDT1, MCM2, and MCM7 onto chromatin). FACS analysis confirmed each phase accordingly. A gradual increase of cyclin K on chromatin was observed, and loading of cyclin K appeared to be in parallel with pre-RC constituents (Fig. 3b). Next, we examined cells synchronized by serum starvation (Fig. 3c). FACS analysis showed that around 90% of cells were synchronized at G0. When released into serum-containing medium, the kinetics of cyclin K loading onto chromatin was again in parallel with pre-RC formation (indicated by Mcm2). It is generally accepted that maximal amount of MCM2-7 proteins are loaded onto chromatin at G1 to S phase transition^3^. Of note, maximal loading of cyclin K happened between 8 and 12 h, coinciding with G1 to S phase transition as demarcated by FACS analysis (Fig. 3c). This notion was further corroborated in cells synchronized by hydroxyurea treatment (Fig. 3d). FACS analysis confirmed expected synchronization of cells in late G1 and early S phase. Release into fresh medium immediately decreased chromatin-bound cyclin K, similar to MCM proteins. Finally, loading was examined using in vivo partial hepatectomy model^25^. Quiescent hepatocytes reentered the cell cycle as indicated by robust labeling of BrdU and Ki67 (Supplementary Fig. 3). Cell lysates were prepared from hepatocytes at indicated time point. Once again, the loading of cyclin K and pre-RC assembly coincided with the entry and progression of the cell cycle (indicated by loading of Mcm2 onto chromatin) (Fig. 3e).

### Cyclin K is required for pre-RC assembly

**Fig. 4:**
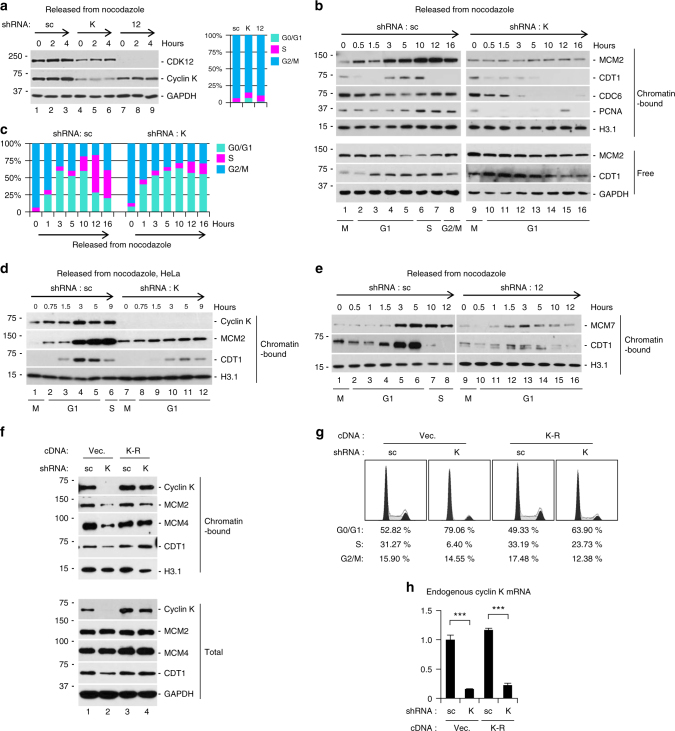
Cyclin K is required for pre-RC assembly. **a** HCT116 cells were knocked down by indicated shRNA for 2 days and synchronized at M phase using procedure described in Fig. 2j, k. A portion of cells were used for protein blotting analysis (left panel), and the rest for FACS analyses (right panel). 0 h denoted cells collected immediately after release into fresh medium without nocodazole (800 ng/ml). **b** Sequential loading of prereplicative complex constituents (CDC6 followed by CDT1 and finally MCM2) onto chromatin in HCT116 cells from **a** were analyzed. Chromatin-bound PCNA marked S phase entry. Corresponding cell cycle phases were labeled at the bottom on basis of FACS analysis in **c**. **c** An aliquot of cells from **b** at each time point were analyzed by FACS analysis to demarcate each cell cycle phase. **d** Similar experiment as in **b** using HeLa cells. **e** Sequential loading of prereplicative complex constituents (CDT1 and MCM7) onto chromatin with or without CDK12 knockdown in HCT116 cells. **f** Rescue of pre-RC assembly defect by shRNA-resistant cDNA encoding cyclin K (K-R). cDNA was transduced into HCT116 cells, followed by transduction of shRNA 12 h later. vec., vector control. **g** Rescue of cell cycle defect by shRNA-resistant cyclin K in HCT116 cells. **h** An aliquot of HCT116 cells from **f** were used to determine the level of endogenous cyclin K transcript by qPCR analysis. Data are means ± SEM (*n* = 3) (***p < 0.001; Student’s *t*-test). All experiments were repeated at least three times and representative results are shown

Concurrence of loading of cyclin K with pre-RC assembly suggested that pre-RC assembly might require cyclin K activity. Cyclin K or CDK12 was knocked down, and cells were synchronized in M phase using the procedure described in Fig. 2j, k (Fig. 4a). To initiate pre-RC assembly, synchronized cells were released into fresh medium. Chromatin-bound and soluble fractions were examined at different time points to monitor the progress of cell cycle^30^. In control cells, CDT1 and MCM2 proteins peaked between 5 and 10 h after release, indicating the completion of pre-RC assembly as well as the end of G1 phase (Fig. 4b, lanes 2–5). Consistently, a simultaneous increase of chromatin-bound PCNA indicated entry into S phase (Fig. 4b, lanes 6–7). FACS analysis correlated cell cycle profiles with the loading of these proteins (Fig. 4c). In contrast, cyclin K knockdown abolished loading of CDT1 and MCM2 onto chromatin (Fig. 4b, lanes 10–16). Initially after release from M phase, the kinetics of CDC6 was comparable with or without knockdown (Fig. 4b, compare lanes 1–3 to 9–11). In control cells, chromatin-bound CDC6 protein increased when cells entered S phase (Fig. 4b, lanes 6–7). Because cells arrested in G1 after cyclin K knockdown, no increase in chromatin-bound CDC6 protein was seen as expected (Fig. 4b, lanes 13–16). Residual loading of PCNA in knockdown cells was likely because a small percentage of cells were not transfected by shRNA construct (Fig. 4b, lane 15). Reduced pre-RC formation by cyclin K knockdown was also observed in another cell line (Fig. 4d), indicating a general phenomenon. Knockdown of CDK12 largely eliminated pre-RC assembly (Fig. 4e), indicating that the effect of cyclin K is kinase-dependent. Rescue experiments were also carried out to further corroborate the effect of cyclin K knockdown. Silent mutations were introduced to cDNA encoding cyclin K (K-R) to disrupt base-pairing by the seeding sequence of shRNA. The level of ectopically expressed, shRNA-resistant cyclin K on chromatin was comparable to that in control cells (upper panel, Fig. 4f). Accordingly, loading of pre-RC components (CDT1, MCM2, and MCM4) was restored (upper panel, Fig. 4f). In addition, the cell cycle defect caused by cyclin K knockdown was also largely rescued (Fig. 4g). Both endogenously and ectopically expressed cyclin K have the same molecular weight, thus cannot be separated on SDS-PAGE gel. To ensure that cyclin K-shRNA was also functional at the presence of shRNA-resistant cDNA construct (K-R), the mRNA level of endogenous *cyclin K* was determined by qPCR using primers targeting the 3′-UTR region of endogenous *cyclin K* (cDNA construct does not contain this sequence). We found that endogenous cyclin K was knocked down by shRNA to a similar degree with or without expression of the shRNA-resistant cDNA (Fig. 4h). Taken together, our results demonstrate that cyclin K is required for pre-RC assembly.

We explored the underlying mechanisms how cyclin K controlled pre-RC assembly (Supplementary Fig. 4). The pre-RC is formed by sequential recruitment of ORC1–ORC6, CDC6, CDT1, and finally MCM2–MCM7^3^. As shown in Fig. 4b, cyclin K knockdown prevented loading of CDT1 and subsequently MCM proteins in G1 without affecting CDC6 kinetics in G1. Since correct loading of the ORC complex is a prerequisite for CDC6 loading, we expected that cyclin K knockdown would not affect ORC. Indeed, loading of ORC constituents (ORC4 and ORC6) was comparable between control and knockdown cells (Supplementary Fig. 4a). Next, we examined whether the regulation of CDT1 was abnormal upon cyclin K knockdown. It is well established that Geminin protein (encoded by *GMNN*) accumulates during M phase to ensure accumulation of sufficient amount of CDT1 protein for licensing at following G1. On the other hand, Geminin protein is quickly degraded when cells enter G1 to allow CDT1 binding to chromatin^31^. We therefore asked whether defect in CDT1 loading was caused by perturbation of Geminin protein. As shown in Supplementary Fig. 4b, both accumulation and degradation kinetics of Geminin protein was similar with or without cyclin K knockdown. Cyclin B1 protein was also degraded at a comparable rate. CDT1 activity is tightly controlled by proteasome^6^. Treatment with proteasome inhibitor increased the level of free CDT1 to a similar degree in both control and knockdown cells, but did not restore chromatin-bound CDT1 levels induced by cyclin K knockdown (Supplementary Fig. 4c). We concluded that the defect of pre-RC assembly was unlikely caused by abnormal mitotic exit or dysregulation of CDT1 protein stability.

We considered the possibility that knockdown of cyclin K might induce a global defect in chromatin structure or transcription to impede loading of pre-RC constituents. To rule out this possibility, we examined the loading kinetics of several protein complexes with or without cyclin K knockdown (Supplementary Fig. 4d). Loading of RNA polymerase II onto chromatin appeared to be comparable. In addition, Ser5 phosphorylation of the C-terminal domain of RNA polymerase II was not affected, indicating normal recruitment of TFIIH protein kinase complex for transcription initiation^32^. Loading of CDK9 protein, the major kinase controlling transcription elongation^33^, was similar with or without cyclin K knockdown. Loading of SMARCA4, a constituent of SWI/SNF chromatin remodeling complex^34^, was also not affected. Previous studies suggested that CDK12/cyclin K promotes transcription elongation because it could phosphorylate Ser2 of the C-terminal domain of RNA polymerase II. Nevertheless, these studies also reported that there was no change in global transcription upon CDK12 or cyclin K knockdown^19,20^. To test whether block on transcription elongation could have an effect on pre-RC loading, we treated cells with actinomycin D (ActD), a well-known global inhibitor of RNA polymerase II transcription elongation. M phase-synchronized cells were treated with or without ActD for 15 min before being released into fresh medium. Fresh medium did not contain nocodazole to allow cell cycle progression into G1, but still contained ActD to allow continuous block of RNA polymerase II transcription elongation. After 5 h, cells were collected for FACS as well as protein blotting analyses (Supplementary Fig. 4e). As expected, prolonged ActD treatment greatly reduced chromatin-bound RNA polymerase II. However, neither cell cycle entry into G1 (indicated by FACS analysis), degradation of cyclin B1 protein, nor loading of pre-RC components (MCM2 and MCM4) was affected by ActD treatment (Supplementary Fig. 4e). We also tested adding ActD immediately after release into cell cycle, and obtained similar results. Similarly, other global transcription inhibitors did not have an overt effect on pre-RC loading, including THZ1 to inhibit transcription initiation by targeting CDK7, and flavopiridol to inhibit transcription elongation by targeting CDK9 (Supplementary Fig. 4f). MYC is well known for its ability to regulate global transcription^35^. Recent studies also showed that knockdown of cyclin K may affect 3′ end processing of c-MYC transcript^20^. We found that cyclin K knockdown only slightly reduced c-MYC protein level. In addition, c-MYC inhibitor treatment did not affect loading of the final pre-RC component (Supplementary Fig. 4g). These observations suggested that pre-RC formation defect induced by cyclin K knockdown was unlikely to be caused by transcription dysregulation, although we could not completely rule out the possibility that cyclin K might regulate transcription of an unknown factor that controls pre-RC assembly. Previous studies showed that prolonged knockdown of cyclin K induced DNA damage in a fraction of cells indicated by γH2AX signals^19^. However, we did not observe obvious γH2AX signals in our experiments (Fig. 2i and Supplementary Fig. 2f), arguing against the possibility that activated DNA damage signaling might be responsible for pre-RC loading defect. Accordingly, treatment with small-molecule ATM and ATR inhibitors failed to reverse the defect in CDT1 loading caused by cyclin K knockdown (Supplementary Fig. 4h). Occasionally we found γH2AX signals in less than 10% of cells at day 5 following knockdown, consistent with the notion that reduced pre-RC assembly eventually leads to DNA damage^2,36^. Taken together, we concluded that the defect in pre-RC assembly is not caused by DNA damage, dysregulation of CDT1 or Geminin proteins, abnormal mitotic exit, global perturbation of chromatin or transcription.

### Cyclin K counteracts cyclin E1 to promote pre-RC assembly

**Fig. 5:**
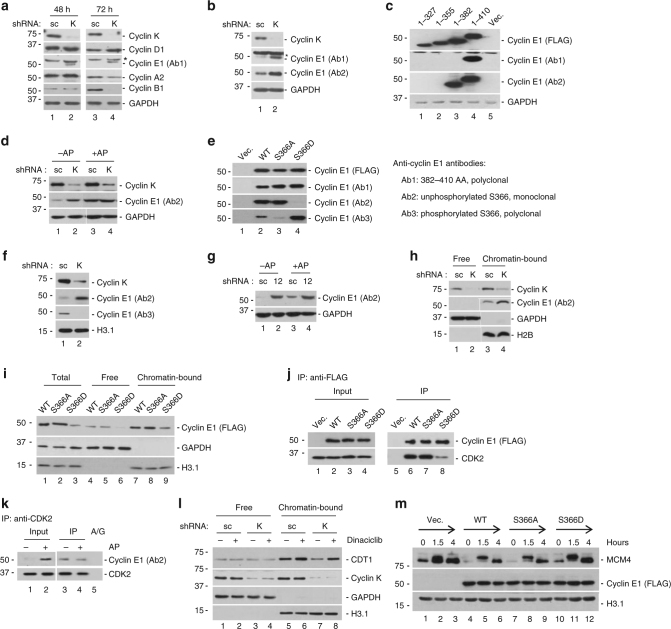
Cyclin K counteracts cyclin E1 to promote pre-RC assembly. **a** Expression profiling of cyclin proteins with or without cyclin K knockdown for 48 and 72 h in HCT116 cells. Ab1 denoted a commonly used commercial anti-cyclin E1 polyclonal antibody. Asterisk denoted the non-specific signal detected by several batches of Ab1. **b** Verification by a monoclonal anti-cyclin E1 antibody (Ab2). Note that Ab2 only detected the faster mobility band of cyclin E1. **c** Demarcation of epitope regions of Ab1 and Ab2 by a series of cyclin E1 truncation mutants. 1–410 denoted full-length cyclin E1. All proteins were FLAG-tagged at the C-terminus, and expression constructs were transfected into HEK293 cells. **d** Alkaline phosphatase (AP) treatment of HCT116 cell lysates derived from cells with or without cyclin K knockdown, followed by protein blotting analyses. **e** Differential recognition of phosphorylation mimics by Ab2 and Ab3. S366A, alanine substitution of serine to mimic unphosphorylated state. S366D, aspartate substitution of serine to mimic phosphorylated state. Expression constructs were transfected into HEK293 cells. Right panel, summary of the specificity of antibodies used in Fig. 5. **f** Endogenous cyclin E1 protein detected by Ab2 and Ab3 with or without cyclin K knockdown in HCT116 cells. **g** Similar experiment as in **d** except that CDK12 (12) was knocked down in HCT116 cells. **h** Detection of endogenous chromatin-bound cyclin E1 by Ab2 with or without cyclin K knockdown in HCT116 cells. **i** Distribution of wild-type and mutant cyclin E1 proteins in soluble and chromatin-bound fractions derived from HCT116 cells. **j** Endogenous CDK2 in HEK293 cells was immunoprecipitated by wild-type or mutant cyclin E1 proteins, followed by protein blot analyses. **k** Endogenous CDK2 in HCT116 cells was immunoprecipitated by anti-CDK2 antibody. Immunoprecipitates were then treated with or without alkaline phosphatase (AP), and associated endogenous cyclin E1 was analyzed. A/G, protein A/G beads without anti-CDK2 antibody. **l** Small-molecule inhibitor of CDK2 (dinaciclib, 500 nM) partially rescued CDT1 loading defect caused by cyclin K knockdown in HCT116 cells. **m** S366A and wild-type but not S366D cyclin E1 prevented pre-RC assembly (indicated by MCM4 loading onto chromatin) in HEK293 cells. All experiments were repeated at least three times and representative results are shown

Time point experiments revealed that G1 arrest commenced between 2 and 3 days after shRNA delivery (Fig. 2f). As aforementioned, cyclin K protein level was expected to decrease 1.5 days post shRNA delivery due to its long protein half-life (Fig. 1f). We thus examined expression levels of cell cycle-related cyclin proteins at early time point. Consistent with G1 arrest starting from day 2 (Fig. 2f), cyclin A2, B1, and D1 protein levels were similar with or without cyclin K knockdown at this point (Fig. 5a). After 3 days, cyclin D1 protein increased likely because more and more cells were arrested at G1. In contrast, cyclin A2 and B1 proteins started to decrease likely because cell cycle could not progress into G2 and M to accumulate them (Fig. 5a). Interestingly, a commonly used commercial anti-cyclin E1 polyclonal antibody (Ab1) detected a band slightly above 50 kD (denoted by asterisk), and an additional band slightly below 50 kD upon cyclin K knockdown at both time points (Fig. 5a). We tested different batches of Ab1 from the same commercial vendor, and only the lower band could be repeatedly seen (Supplementary Fig. 5a), indicating that the upper band was not cyclin E1. In addition, we generated *cyclin E1* knockout cell lines (*Cyclin E1−/−*) using CRISPR/Cas9 genome editing technology. In these cell lines, the lower band was undetectable (Supplementary Fig. 5b). For further verification, we raised a monoclonal antibody against the peptide derived from the C-terminus of human cyclin E1 (Ab2). Ab2 only recognized the lower band (Fig. 5b, Supplementary Fig. 5a, b). These results demonstrated that the lower band was indeed cyclin E1. Thus, cyclin K knockdown induced cyclin E1 accumulation. Of note, cyclin E1 protein level was increased at day 2 before G1 arrest commenced, indicating a causative effect of cyclin E1 protein accumulation on defective pre-RC assembly.

Because high cyclin E1 activity in G1 is known to be detrimental for pre-RC assembly even in highly transformed cancer cells^8^, we set out to investigate the mechanism of cyclin E1 accumulation induced by cyclin K knockdown. Using a series of C-terminal truncation mutants, we mapped the epitope region recognized by polyclonal Ab1 to be amino acids 382–410, and that of monoclonal Ab2 to be amino acids 355–382 of cyclin E1 (Fig. 5c). Interestingly, alkaline phosphatase treatment (AP) largely increased the signal recognized by Ab2 in control cells (Fig. 5d), indicating that Ab2 recognizes cyclin E1 (355–382) in its unphosphorylated state. In cyclin K knockdown cells, alkaline phosphatase treatment did not further increase signal intensity, indicating that most cyclin E1 was unphosphorylated. We substituted all serine (S) and threonine (T) residues within this region individually to either alanine (A) or negatively charged amino acids (D or E) to block or mimic phosphorylation, respectively. As expected, Ab1 recognized all mutant proteins. In contrast, Ab2 detected all but S366D, indicating that phosphorylation at S366 blocks epitope recognition (Fig. 5e and Supplementary Fig. 5c). To confirm this phosphorylation event, we also generated a phospho-specific antibody (Ab3) targeting phosphorylated S366 (Fig. 5e). Knockdown of cyclin K increased the signal by Ab2 while diminished that by Ab3 (Fig. 5f). Similarly, knockdown of CDK12, the endogenous cognate kinase of cyclin K, also increased the signal by Ab2 (Fig. 5g). Thus, knockdown of cyclin K leads to the accumulation of cyclin E1 with unphosphorylated S366.

We investigated the biological implications of S366 phosphorylation. Knockdown of cyclin K enhanced unphosphorylated state of endogenous cyclin E1 on chromatin (Fig. 5h). We considered the possibility that S366 phosphorylation might inhibit binding of cyclin E1 onto chromatin. However, this was unlikely the case because S366A, S366D and wild-type cyclin E1 proteins exhibited similar binding affinity to chromatin (Fig. 5i). FLAG-tagged wild-type and S366A pulled down endogenous CDK2 protein with similar efficiency. In contrast, S366D substitution largely eliminated this interaction (Fig. 5j). We further examined the significance of S366 phosphorylation on endogenous proteins. Endogenous CDK2 and its associated proteins were immunoprecipitated, followed with or without alkaline phosphatase treatment (AP). AP treatment of total cell lysate significantly increased signal by Ab2, indicating that endogenous cyclin E1 protein was phosphorylated at S366 (Fig. 5k). In sharp contrast, AP treatment did not enhance the signal in CDK2-bound cyclin E1 (Fig. 5k), strongly indicating that S366 phosphorylation in endogenous cyclin E1 blocked its interaction with CDK2. Association with CDK2 is known to increase cyclin E1 protein stability. Indeed, we found that cyclin E1 accumulated to a much less degree in *CDK2* knockout cells (Supplementary Fig. 6). We reasoned that this enhanced CDK2-cyclin E1 interaction induced by cyclin K knockdown might contribute to blocking pre-RC formation. Indeed, CDK2 kinase activity was increased by cyclin K knockdown (Supplementary Fig. 7), and pharmaceutical inhibition of CDK2 by dinaciclib largely restored pre-RC assembly when cyclin K was knocked down (Fig. 5l). As dinaciclib might also inhibit CDK1/cyclin B1, it was added to the cell culture at 3 h following release from the M phase. At this time point, cyclin B1 protein level was barely detectable, and therefore CDK1 should not be active. In addition, ectopic expression of cyclin B1 did not inhibit pre-RC assembly (Supplementary Fig. 8). Thus, we concluded that the rescue effect of dinaciclib was not due to CDK1 inhibition. Ectopic expression of cyclin E1 prevents pre-RC assembly even in highly transformed cancer cells^8^. Consistently, S366A and wild-type cyclin E1 proteins largely blocked pre-RC formation whereas S366D failed to do so (Fig. 5m). Of note, ectopically expressed wild-type cyclin E1 protein could be detected by both Ab2 and Ab3, indicating incomplete phosphorylation at S366 by cyclin K/CDK12 (Fig. 5e). Thus, cyclin K/CDK12 directly or indirectly maintains cyclin E1 S366 phosphorylation to block its interaction with CDK2 during pre-RC assembly in early G1. Without interacting with CDK2, cyclin E1 is quickly degraded by proteasome in cells.

### Uncontrolled CDK2 activity in G1 inhibits pre-RC assembly

**Fig. 6:**
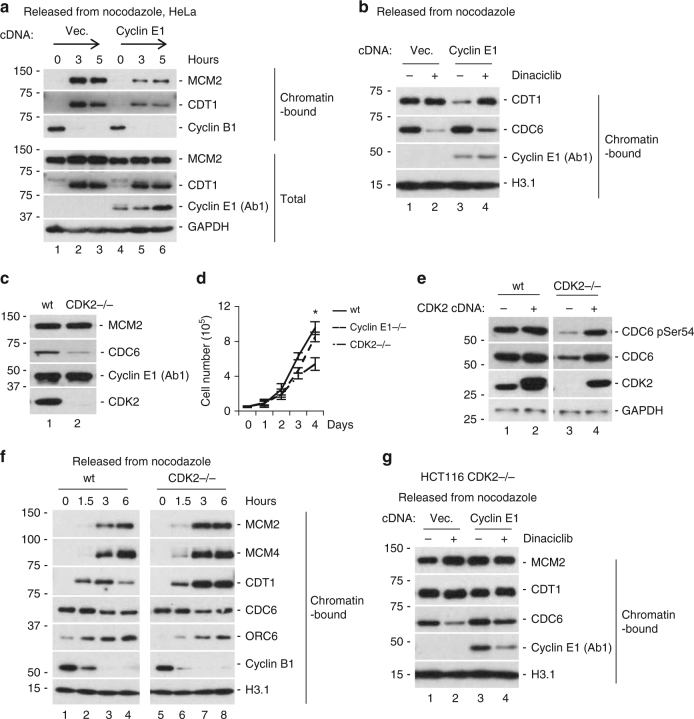
Uncontrolled CDK2 activity in G1 inhibits pre-RC assembly. **a** Ectopic expression of cyclin E1 inhibited pre-RC formation in various cell lines. HeLa cells were transfected with vector control (vec.) or cDNA encoding cyclin E1 (Cyclin E1). Cells were synchronized at M phase and released using the same protocol in previous figures. Similar results were obtained in HEK293 (Fig. 5m) and HCT116 cells (**b**). **b** CDK2 inhibitor treatment (dinaciclib, 500 nM) eliminated the negative effect of cyclin E1 on pre-RC assembly. HCT116 cells were transduced with vector control (vec.) or cDNA encoding cyclin E1 (Cyclin E1). Cells were synchronized at M phase and released into fresh medium with or without small-molecule CDK2 inhibitor. **c** CDC6 protein level was reduced in *CDK2* knockout HCT116 cell lines. Similar results were obtained in several independent cell lines. Knockout cell lines were generated by CRISPR/Cas9 technology. **d** Growth curve comparison of wild-type (wt), *cyclin E1* knockout (*Cyclin E1−/−*) and *CDK2* knockout (*CDK2−/−*) HCT116 cell lines. Data are means ± SEM (*n* = 3) (*p < 0.05; Student’s *t*-test; *CDK2−/−* compared to wt). **e** CDK2 overexpression did not increase CDC6 protein level. Ectopically expressed CDK2 was functional because it restored CDC6 protein level in *CDK2* knockout HCT116 cells. **f** The kinetics of pre-RC assembly was comparable in wild-type and *CDK2* knockout HCT116 cells. **g** *CDK2* knockout eliminated the negative effect of cyclin E1 on pre-RC assembly, consistent with CDK2 inhibitor treatment (**b**). All experiments were repeated at least three times and representative results are shown

So far, our results indicated that cyclin K promoted pre-RC assembly by restricting cyclin E1/CDK2 activity. This working model predicted that the effect of cyclin E1 overexpression on pre-RC assembly would be dependent on CDK2, a hypothesis yet to be tested. Consistent with previous studies^8^, we confirmed that ectopic expression of cyclin E1 indeed inhibited pre-RC formation in various cell lines (Figs. 5m, 6a, b). Of note, cyclin E1 overexpression blocked loading of CDT1 and subsequently MCM proteins onto chromatin (Fig. 6a), similar to that of cyclin K knockdown (Fig. 4b). Like cyclin K knockdown (Fig. 4b), it did not affect the amount of CDC6 on chromatin (Fig. 6b). Strikingly, CDK2 inhibitor treatment (dinaciclib) eliminated the effect of cyclin E1 overexpression, and completely restored CDT1 and MCM loading (Fig. 6b). It should be noted that cyclin E1 overexpression did not increase chromatin-bound CDC6. On the other hand, treatment with CDK2 inhibitor greatly reduced chromatin-bound CDC6 although loading of CDT1 was not affected (Fig. 6b). These observations demonstrated that CDC6 was not a limiting factor for pre-RC assembly in cells. Reduced CDC6 protein level by CDK2 inhibitor treatment was consistent with previous findings that CDK2 phosphorylated Ser54 of CDC6 to maintain its protein stability^37^. We generated *CDK2* knockout cell lines (*CDK2−/−*), and confirmed that CDC6 protein level was indeed reduced (Fig. 6c). These cells grew normally albeit with a moderately slower proliferation rate than wild-type or cyclin E1 knockout cells (Fig. 6d). Reintroduction of CDK2 in CDK2 knockout cells fully restored CDC6 protein level to a similar degree to that in wild-type cells (Fig. 6e). It should be noted that overexpression of CDK2 did not increase CDC6 expression (compare lane 1 with 2, Fig. 6e). Thus, it should not be surprising that cyclin K knockdown increased CDK2 activity without increasing total or chromatin-bound CDC6. Although CDC6 was greatly reduced in *CDK2* knockout cells (Fig. 6c), the loading kinetics of pre-RC components was comparable with that in wild-type cells (Fig. 6f). Similar to pharmaceutical inhibition of CDK2 in wild-type cells (Fig. 6b), genetic ablation of *CDK2* rendered cells insensitive to the effect of cyclin E1 overexpression on pre-RC assembly (Fig. 6g). Thus, increased cyclin E1/CDK2 activity in early G1 inhibits pre-RC formation at the step of CDT1 and MCM loading, similar to the defect induced by cyclin K knockdown.

### Cyclin K expression positively correlates with tumorigenesis

**Fig. 7:**
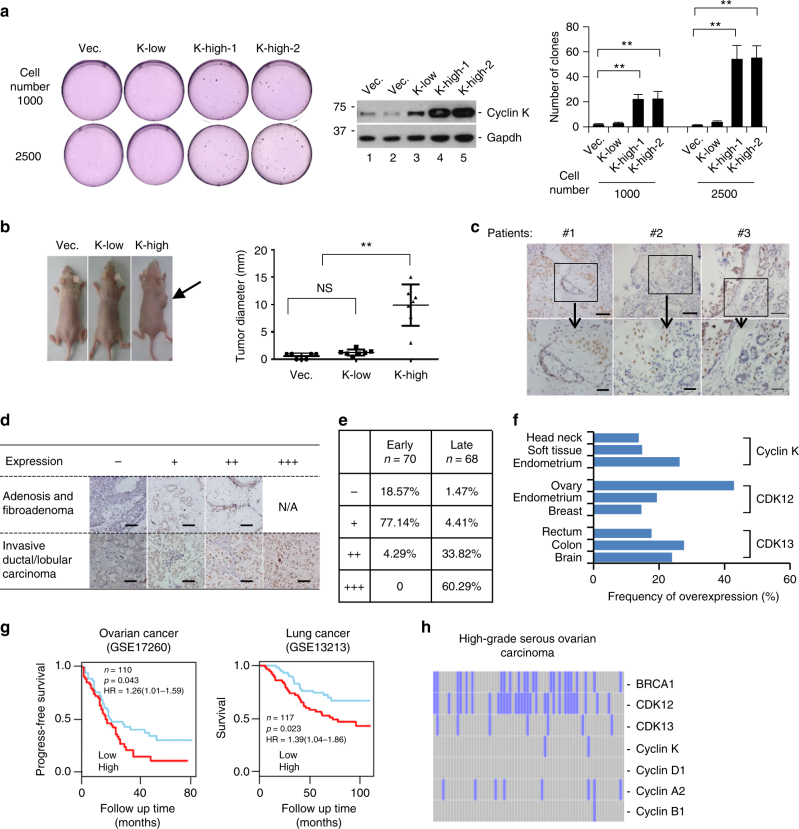
Cyclin K expression positively correlates with tumorigenesis. **a** Ectopic cyclin K expression promoted anchorage-independent growth of NIH 3T3 cells in soft-agar assay. vec., vector. K-low, a cell line stably expressing cyclin K moderately above the endogenous level. K-high-1, K-high-2, two independent cell lines stably expressing high level of cyclin K. 1000 or 2500 cells of each cell line were seeded. Quantification was from four biological replicas. Data are means ± SEM (*n* = 4) (**p < 0.01; Student’s *t*-test). **b** Representative images of the ability of indicated cell lines to promote tumorigenesis in immunocompromised mice. K-high, representative image from both K-kigh-1 and 2 cell lines. More than ten mice were injected with individual cell line. Data are means ± SEM (*n* = 3) (**p < 0.01; Student’s *t*-test). **c** Pilot study of cyclin K expression in human invasive breast ductal carcinoma. Immunochemistry was carried out in paraffin-fixed specimens with a previously characterized anti-cyclin K antibody^22^. Lower panel, enlarged images of boxed areas in the upper panel. Scale bar, 100 μm for upper panel and 40 μm for lower panel. **d** Examination of cyclin K expression in our patient cohort containing early stage (upper panel, 70 patients), and late stage of breast carcinoma (lower panel, 68 patients). −, +, ++, +++, semi-quantification of cyclin K expression level by three trained pathologists at two different clinics (+++ denotes the highest expression of cyclin K). Scale bar, 100 μm. **e** Quantification of **d**. **f** Examination of CDK12, CDK13 and cyclin K expression in various human tumors. Expression data were extracted from COSMIC (Catalog of Somatic Mutations in Cancer, http://cancer.sanger.ac.uk/cosmic). **g** Kaplan–Meier plot of survival in ovarian and lung cancer patients with low or high expression of cyclin K. **h** Expression analyses of indicated genes in 66 cases of cyclin E1-overexpressing high-grade serous ovarian carcinoma. Expression data were extracted from COSMIC. Note that previous studies established that BRCA1 was required for continuous proliferation of cyclin E1-overexpressing cells^47^

Dysregulation of pre-RC assembly via ectopic expression of licensing proteins promotes cellular transformation and tumorigenesis^2^. We hypothesized that cyclin K overexpression might have similar effects. We generated NIH 3T3-based cell lines stably expressing various levels of cyclin K (Fig. 7a). Two independent cell lines with high cyclin K expression, K-high-1, and K-high-2, promoted anchorage-independent growth as well as tumorigenesis in immunocompromised mice (Fig. 7a, b). In contrast, the cell line with low cyclin K expression, K-low, failed to support anchorage-independent growth or tumorigenesis (Fig. 7a, b).

We further investigated the implications of cyclin K overexpression and its relationship with cyclin E1 in patient cohorts. In our pilot study, we examined cyclin K expression in human invasive breast ductal carcinoma. Consistent with its role in promoting cell proliferation, cyclin K was highly expressed only in cancerous cells (Fig. 7c). We extended this observation in a larger patient cohort containing 70 cases of early-stage tumorigenesis (adenosis and fibroadenoma) and 68 cases of late-stage human breast cancer specimens (invasive ductal/lobular carcinoma). Semiquantitative analyses of cyclin K immunostaining were carried out by independent pathologists at two clinical sites (Fig. 7d). Higher cyclin K expression was much more prevalent in late-stage than early-stage of tumorigenesis (Fig. 7e). Overexpression was also observed in various tumor types with frequency ranging from 15 to 40% (Fig. 7f). Consistently, higher expression of cyclin K correlated with lower survival rate in patients (Fig. 7g). Cyclin E1 overexpression is detrimental for pre-RC assembly, yet paradoxically it is frequently seen in highly proliferative human tumors. Our results aforementioned imply that cyclin K restricts the activity of cyclin E1 during pre-RC assembly in G1. We thus hypothesized that in tumors with cyclin E1 overexpression, cyclin K and its cognate kinases CDK12 and CDK13 might also be overexpressed in order to counteract upregulated cyclin E1 activity in early G1. Indeed, this was the case in highly proliferative, cyclin E1-overexpressing human high-grade serous ovarian carcinoma (Fig. 7h).

## Discussion

The assembly of pre-RC (licensing) is a prerequisite for DNA replication and sustained cell proliferation^3^. Our data establish cyclin K as an essential factor to promote efficient licensing in G1 phase (Supplementary Fig. 9). This observation provides plausible explanations for phenomena associated with cyclin K and its cognate kinases in current literature. Genetic ablation of *cyclin K* in mice results in preimplantation lethality^19^, and *CDK12* knockout also leads to early embryonic lethality albeit shortly after implantation^38^. We suspect that these phenotypes are caused by lack of the ability to assemble pre-RC in *cyclin K*-null or *CDK12*-null embryonic cells. Reduced licensing inevitably leads to DNA damage in cells eventually^7^, a phenomenon also observed in *CDK12*-null embryos as well as in cancer cells with cyclin K knockdown^19,38^. It should be noted that short-term knockdown of cyclin K or CDK12 did not elicit overt DNA damage on our hands. We and others recently established that cyclin K interacts with CDK12 and CDK13 in cells^19,23,38^. Slightly delayed lethality in *CDK12* knockout mice may be due to a partial compensation by CDK13. Of note, our results do not exclude the possibility that CDK13 may also regulate pre-RC assembly. We tested commercial anti-CDK13 antibodies from several commercial sources, but failed to detect robust CDK13 expression by immunoblotting in a dozen of cancer cell lines tested. This may indicate that CDK13 expression is very low, and thus does not contribute significantly to pre-RC assembly in these cells. We previously showed that cyclin K expression was the highest in embryonic stem cells (ESCs), and required to maintain self-renewal in ESCs^23^. Interestingly, a recent study demonstrated that ESCs functionally require more pre-RCs than their differentiated derivatives^39^. Licensing proteins are oncogenic, and can be used as histopathological markers of proliferation capacity^2^. Likewise, we find that cyclin K expression is much higher in aggressive tumors in our patient cohort. Therefore, the role of cyclin K in the regulation of pre-RC assembly likely explains why its expression is highly correlated with cell proliferative state under both developmental and pathological contexts.

Here, we uncover a novel phosphorylation event at S366 of cyclin E1, and this phosphorylation depends on cyclin K and CDK12. Cyclin E1 is frequently overexpressed in fast-proliferating cancer cells, consistent with its established role in promoting S phase progression. Paradoxically, overexpression of cyclin E1 is also deleterious for pre-RC formation even in highly transformed cancer cells^8^. This strongly indicates that there must be a mechanism to restrict cyclin E1 activity in G1 phase. Classical endogenous CDK inhibitors such as p27 are unlikely the solution. Unlike cyclin K knockdown, deletion of these inhibitor proteins usually leads to cell cycle acceleration rather than G1 arrest^40^. Our results demonstrate that cyclin K plays a key role to restrict the detrimental activity of cyclin E1 in G1 phase. This notion predicts that tumor cells overexpressing cyclin E1 should need high level of CDK12 and/or cyclin K to sustain high rate of proliferation. Indeed, we found that this is the case in many tumors in COSMIC database. Of note, recent whole-genome sequencing studies of human high-grade serous ovarian carcinoma (HGSC) indicate that cyclin E1 overexpression is the driving force of tumor initiation and progression in about 30% of patients^13,14^. HGSC is the deadliest gynecological cancer worldwide with very limited treatment options^41^. We found that a large portion of HGSC with high cyclin E1 expression also exhibit high expression of CDK12 or cyclin K. It will be of great interest to determine whether HGSC with cyclin E1 amplification are more sensitive to newly discovered CDK12 chemical inhibitors^42^. Interestingly, the same whole-genome sequencing study also identified heterozygous *CDK12* mutations in less than 2% of HGSC^14^. Some of these mutations likely weaken the interaction with cyclin K^43^. Knockdown of CDK12 or cyclin K to 50% of the endogenous level (to mimic the effect of heterozygous mutation) did not cause a global defect in pre-RC assembly. However, we cannot rule out the possibility that moderately reduced CDK12 activity caused by those heterozygous mutations could gradually cause insufficient pre-RC formation in a long run, eventually leading to genomic instability. Indeed, reduced pre-RC assembly in mice via downregulation of licensing proteins leads to tumorigenesis^36,44,45^. Nevertheless, whenever dysregulated, CDK12 is overexpressed rather than mutated in vast majority of human cancers, highlighting its important role in promoting cell proliferation.

To our knowledge, present study is the first to establish a direct role of cyclin K in the regulation of DNA replication. Recent studies by other groups also indicate that cyclin K and CDK12 may regulate RNAP II transcription^19–21,46^. Together, these studies suggest an exciting scenario that cyclin K may mediate the crosstalk between DNA replication and transcription, a coordinated event in cell cycle that is poorly understood. More importantly, our results suggest a new strategy to treat cyclin E1-overexpressing tumors. Of note, cyclin K stood out as one of the top candidates in unbiased screening studies to be required for continuous cell proliferation in cyclin E1-overexpressing cells^47^. Because compensatory mechanisms exist in cells for loss of CDK2/cyclin E activity^48^, inhibition of CDK12 and/or CDK13 may be a more effective approach to treat deadly cancers such as high-grade serous ovarian carcinoma.

## Methods

### Plasmids

shRNA targeting genes of interest were cloned into pLKO.1 vector (Addgene): Cyclin K-1: GCAGGACGTTTGTGCAAATTT, Cyclin K-2: CCACCAAATCCTGGATCTTTA, CDK12: GCACTGAAAGAGGAGATTGT. Knockdown efficiency was determined by qPCR as well as protein blotting. Human cyclin E1 and CDK2 cDNA were cloned from total RNA extracted from HCT116 cells, and identical to *CCNE1* (Gene ID: 898) and *CDK2* (Gene ID: 1017) in NCBI. Amino acid substitution was generated by site-directed mutagenesis. To generation *CDK2* and *cyclin E1* knockout cell lines, sgRNAs were designed using CRISPRtool (http://crispr.mit.edu) to minimize potential off-target effects. Knockout cell lines were verified by absence of corresponding proteins on protein blot, and by Sanger sequencing of genomic regions targeted by sgRNAs. Primer and oligo sequences are listed in Supplementary Table 1.

### Antibodies and protein blotting

Following commercial antibodies were used: anti-FLAG M2 (Sigma, a8562, 1:20,000 dilution), anti-Sox2 (Santa Cruz, sc-20088, 1:500 dilution), cyclin D1 (sc-753, 1:500 dilution), cyclin E1 (sc-198, 1:500 dilution), cyclin A2 (sc-596, 1:1000 dilution), cyclin B1 (sc-245, 1:1000 dilution), CDK2 (sc-163, 1:5000 dilution), CDK9 (sc-98491, 1:2000 dilution), CDK12 (sc-81834, 1:500 dilution), ORC6 (sc-390490, 1:500 dilution), MCM4 (sc-22779, 1:5000 dilution), MCM7 (sc-9966, 1:1000 dilution), CDT1 (sc-28262, 1:1000 dilution), CDC6 (sc-9964, 1:500 dilution), PCNA (sc-56, 1:5000 dilution), Geminin (sc-13015, 1:500 dilution), p53 (sc-6243, 1:500 dilution), SMARCA4 (sc-25931, 1:1000 dilution) and p21 (sc-397, 1:500 dilution), anti-Pol II (Covance, 8WG16, MPY-127R, 1:2500 dilution) and Ser5-CTD (Covance, H5, MPY-129R, 1:2500 dilution), anti-GAPDH (KangChen, KC-5G5, 1:20,000 dilution), anti-MCM2 (Zen BioScience, 220023, 1:5000 dilution) and SF3B1 (612452, 1:2500 dilution), anti-MYC (Abcam, ab32072, 1:10,000 dilution), H2B (ab1790, 1:2500 dilution) and H3.1 (P30266M, 1:100,000 dilution), anti-CDC6 pSer54 (HuaAn Biotechnology, ET1612-96, 1:2000 dilution), anti-phospho-T/S-Pro (Abcam, ab9344, 1:1000 dilution). Anti-cyclin K antibody was previously described^22,23^. Generation of in-house anti-cyclin E1 antibodies. Monoclonal antibody was produced using fragment derived from human cyclin E1 containing amino acid residue 307–410 (Ab2). The exact epitope was determined to be 355–382 amino acids containing unphosphorylated S366 as described in the text. Polyclonal antibodies were generated using synthetic peptide CNIQTHRD(p-Ser)LDLLDKA (359–373 amino acids of human cyclin E1) (Ab3). The N-terminal cysteine residue was added to conjugate with keyhole limpet hemocyanin. Crude antiserum was incubated at 55 °C for 30 min, and affinity purified using Actigel ADL resin (Sterogene) as previously described^23^. The specificity and epitope was determined by several criteria, including recognition of endogenously and ectopically expressed human cyclin E1, differential recognition of truncation and amino acid substitution cyclin E1 mutant proteins as described in the RESULTS. For protein blotting analyses, at least three biological replicas were carried out, and for each biological replica, two technical replicas were performed. Representative images were shown.

### Cell culture and manipulations

Cancer cell lines were purchased from ATCC, and cultured following ATCC guidelines. Human foreskin fibroblast (HFF) was derived from surgical specimens in West China Second University Hospital. All synchronization procedure was monitored by FACS analysis to confirm cell cycle arrest at specific stages. Synchronization procedure was optimized for each cell line monitored by FACS analysis. M-phase cells were obtained by nocodazole treatment followed by shake-off (>95% purity). To obtain G0-phase cells, NIH 3T3 cells or HFF were cultured in DMEM containing 0.2% FBS (>80% purity). To obtain cells in G1/S transition and early S phase, cells were treated with hydroxyurea (>95% purity). To release from the blockade, cells were spun down, washed in PBS three times, and then plated into fresh standard medium.

### Cell lysate fractionation

Separation of proteins into free and chromatin-bound fractions was performed as previously described^17,30^. Briefly, cells were spun down, resuspended in CSK buffer (0.1% Nonident P-40, 10 mM PIPES [pH 7.0], 100 mM NaCl, 3 mM MgCl_2_ and 300 mM sucrose), incubated on ice for 15 min, and then centrifuged at 2500×*g*/4 °C for 5 min. After removal of supernatant, insoluble fractions were resuspended by SDS-PAGE sample buffer, followed by sonication and centrifugation at 12,000×*g* for 1 min. Supernatant was collected and subjected for following analyses.

### Murine tissue isolation and partial hepatectomy

Murine organs were surgically removed from C57BL/6 mice, rapidly grounded in liquid nitrogen, and transferred to a 1.5 ml tube containing prechilled buffer (25 mM Tris [pH 7.6], 150 mM NaCl, 1% Nonidet P-40, 1% sodium deoxycholate, 0.1% SDS, proteinase inhibitors (Roche), 0.1% PMSF). Lysates were incubated on ice for 30 min, vortexed every 10 min, centrifuged at 17,000×*g*/4 °C for 15 min. Cleared cell lysates were separated by 10% SDS-PAGE, followed by protein blot analyses. Partial hepatectomy procedure was conducted using 6–8-weeks-old C57BL/6 male mice as previously described^25^.

### Immunoprecipitation

Cells were lysed in ice cold buffer (150 mM NaCl, 1.5 mM MgCl_2_, 10 mM KCl, 20 mM Tris [pH 7.9], 0.5 mM EDTA, 10% glycerol, 1 mM DTT, 0.1% PMSF, EDTA-free complete protease inhibitor mixture (Roche) and 0.5% Nonidet P-40) for 15 min. Total cell lysates were cleared by centrifugation at 17,000×*g*/4 °C for 10 min. Supernatant was mixed with indicated antibodies, and rotated for 2 h at 4 °C. Beads were washed by the same buffer for three times (100 beads volume each time). Immunoprecipitates were eluted by 2X SDS-PAGE sample buffer for subsequent analyses. For CDK2 kinase assay, endogenous CDK2 protein was pulled down by anti-CDK2 antibody immobilized on protein A/G agarose beads. Recombinant human histone H1 (Abcam, ab198676) was used as substrate (20 mM HEPES/KOH pH 7.9, 5 mM MgCl_2_, 1 mM DTT and 1 mM ATP). Reactions were terminated by 2X SDS-PAGE sample buffer, and phosphorylated H1 protein was detected by anti-phospho-T/S-Pro (Abcam, ab9344).

### Immunohistochemistry, immunofluorescence and FACS

Paraffin-embedded patient samples were sectioned (3 μm), and mounted on positively charged slides. Staining procedure was previously optimized for anti-cyclin K antibody^22^. Camptothecin treatment (CPT, 2 μM) was used as a positive control for indicated by γH2AX staining (Zen BioScience). Immunofluorescence analysis was carried out as previously described^23^. To profile cell cycle by FACS analysis, cells were fixed in 70% ice cold ethanol, treated with RNase A (0.1 mg/ml), and then stained with propidium iodide (50 μg/ml). More than 20,000 events were collected by BD FACSCalibur and analyzed by ModFit LT software. To determine cell proliferation status, incorporation of EdU was measured following manufacturer’s instruction (Invitrogen). To analyze apoptosis, annexin V staining was used (Invitrogen).

### Anchorage independent growth and in vivo tumorigenicity

Colony formation in soft agar was assayed (*n* > 3) by plating 1000 or 2500 NIH 3T3 cells stably expressing different levels of cyclin K. Top layer contained 0.3% (w/v), and lower layer contained 0.6% (w/v) agar. Plates were incubated for 3–4 weeks, and replenished with fresh medium every 4 days. Colonies were visualized by crystal violet staining. Immunocompromised mice (BALB/c-nu, 6–8 weeks old) were injected subcutaneously with 2 × 10^6^ cells, and monitor every day (*n* > 10 for each cell line). Animal experiments were approved by the Ethical and Animal Welfare Committees of Sichuan University.

### Patient cohort analysis

Informed consent was obtained from all subjects at the time of surgery, and tumor specimen usage was reviewed and approved by the Ethical Committees of Sichuan University as well as Hospital 363 of Aviation Industry Corporation of China. Paraffin-embedded breast cancer specimens were obtained from our hospitals, and graded by highly trained pathologists. Staining procedure was previously optimized for anti-cyclin K antibody^22^. Semi-quantitation of cyclin K expression level was determined by three pathologists, and arbitrarily assigned into four levels. For analyses of expression levels of CDK12, CDK13 and cyclin K in multiple tumor types, data were extracted from COSMIC (Catalog of Somatic Mutations in Cancer, http://cancer.sanger.ac.uk/cosmic). For co-expression analysis in ovarian serous carcinoma, data was extracted from TCGA ovarian serous carcinoma analysis (COSMIC study ID, COSU331). Expression levels of indicated genes were compiled in samples with cyclin E1 overexpression (66 out of 266 cases).

### Statistical analysis

Kaplan–Meier survival curves for different levels of cyclin K in ovarian (GSE17260) and lung (GSE13213) cancer were constructed by Graphpad Prism version 6.01. Quantitation was represented as mean ± S.E.M. All statistical analyses were performed using unpaired two-tailed *t*-test unless otherwise indicated. *p* < 0.05 was considered statistically significant.

### Data availability

The authors declare that all data that support the findings of this study are available within the article and its Supplementary Information files or from the corresponding author on reasonable request.

## Electronic supplementary material

Supplementary Information
