# Supplementary material for: Cyclin K regulates prereplicative complex assembly to promote mammalian cell proliferation
Source: Nat Commun. 2018 May 14;9:1876. doi: 10.1038/s41467-018-04258-w (PMC5951916; doi:10.1038/s41467-018-04258-w)
Supplement: Supplementary file 1 — Supplementary Information [file 41467_2018_4258_MOESM1_ESM.pdf]

Supplementary Information for

Cyclin K regulates prereplicative complex assembly to promote mammalian cell proliferation

Lei et. al

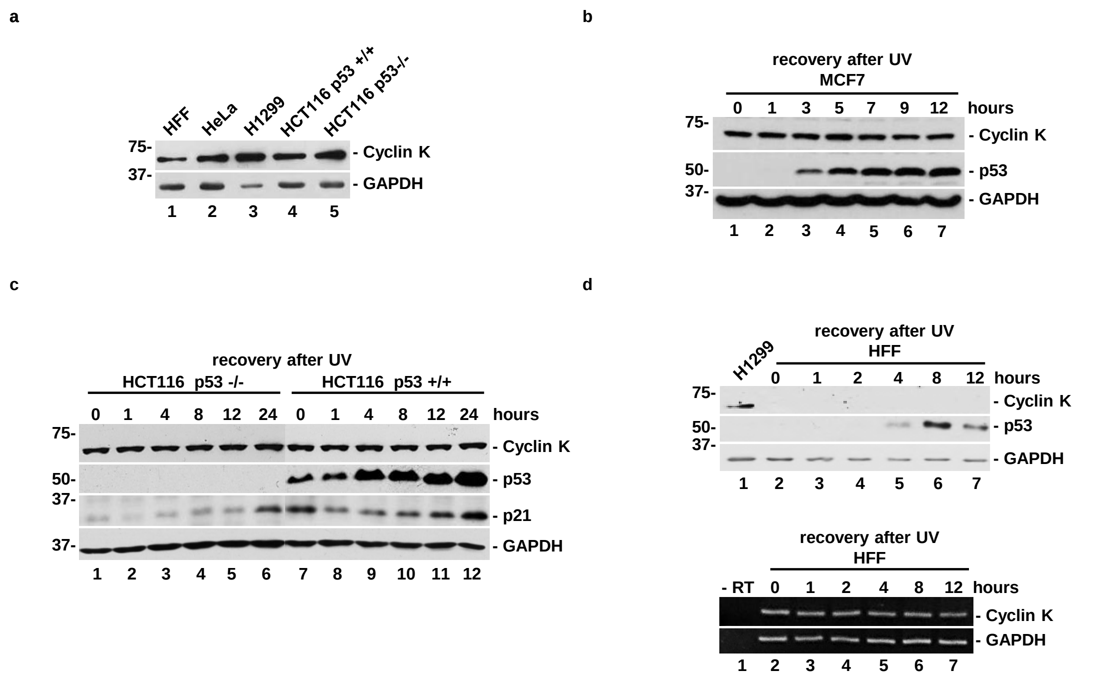

Supplementary Fig. 1: Cyclin K expression is not regulated by p53 protein.

(a) Expression analysis of cyclin K by immunoblotting in various cell lines. HFF, neonatal human foreskin fibroblast. p53 is inactivated in HeLa cells and null in H1299 cells. **(b)** Time course analyses of cyclin K expression by immunoblotting after ultraviolet radiation (UV, 40 J/m^2) in MCF7 cells. **(c)** Time course analyses of cyclin K expression by immunoblotting after UV treatment in isogenic HCT116 colon cancer cell lines with wild-type or knockout of *p53*. **(d)** Time course analyses of cyclin K expression after UV treatment in normal human cells. Upper panel, protein blot analyses. Lower panel, analyses by reverse transcription polymerase chain reaction (RT). HFF, neonatal human foreskin fibroblast. All experiments were repeated three times and representative results are shown.

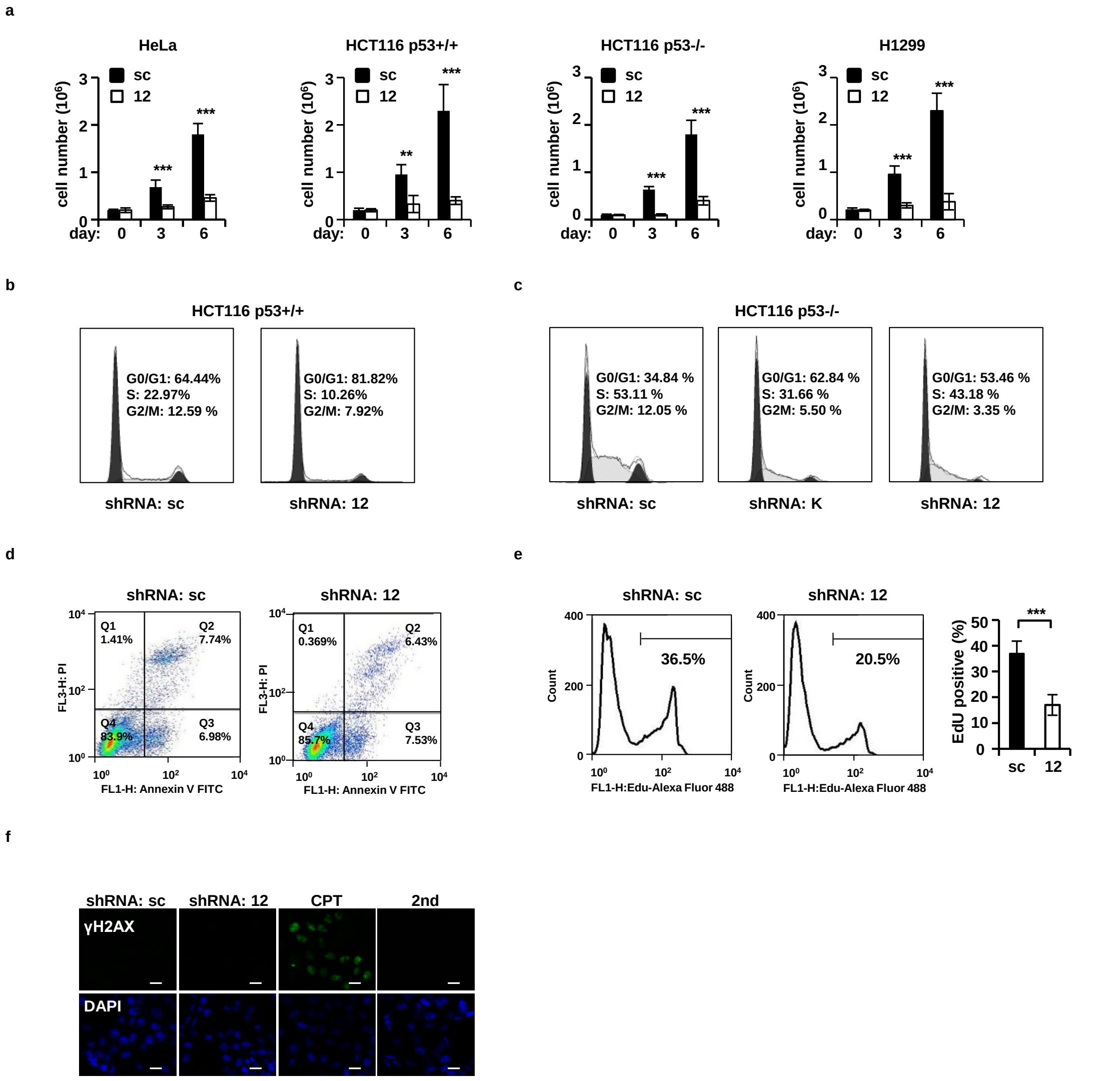

Supplementary Fig. 2: Knockdown of CDK12 reduces cell proliferation, similar to knockdown of cyclin K.

(a) Knockdown of CDK12 reduced proliferation in various human cancer cells. Data are means ± SEM (n = 5) (****p* < 0.001; Student's t-test). **(b)** Cell cycle profiling of HCT116 cells with or without CDK12 knockdown. **(c)** Cell cycle profiling of *p53*-null HCT116 cells with or without CDK12 knockdown. **(d)** The percentage of Annexin V-positive (marker of early apoptosis) HCT116 cells was similar with or without CDK12 knockdown. **(e)** Detection of EdU-positive (marker of proliferation) HCT116 cells by FACS analysis with or without CDK12 knockdown. Quantitation was generated from

five independent experiments. Data are means ± SEM (n = 6) (***$p < 0.001$; Student's t-test). **(f)** Immunofluorescence analysis of γH2AX (marker of DNA damage) in HCT116 cells with or without CDK12 knockdown. CPT was used as a positive control for DNA damage induction. Scale bar, 15 μm. All experiments were repeated at least three times and representative results are shown.

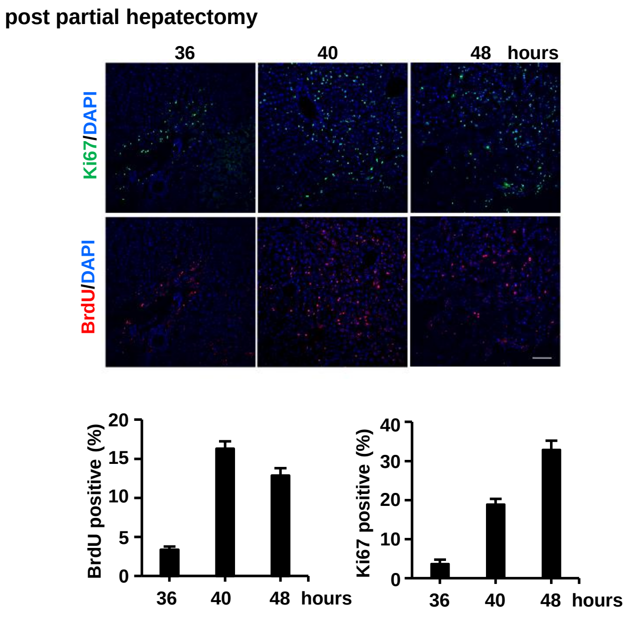

Supplementary Fig. 3: Cell cycle reentry after partial hepatectomy.

Two hours before sacrifice, BrdU was injected into mice. BrdU was used to mark cells in the S phase, and Ki67 to label proliferating cells. Results were quantified from four mice at each time point. The differences on the time point of peak detection for BrdU and Ki67 were likely because BrdU labelling only detected proliferative cells in a 2-hour timeframe, while Ki67 detected all proliferative cells accumulated in the past 48-72 hours. The kinetics of cyclin K and Mcm2 accumulation mimicked that of Ki67. Scale bar, 100 μm.

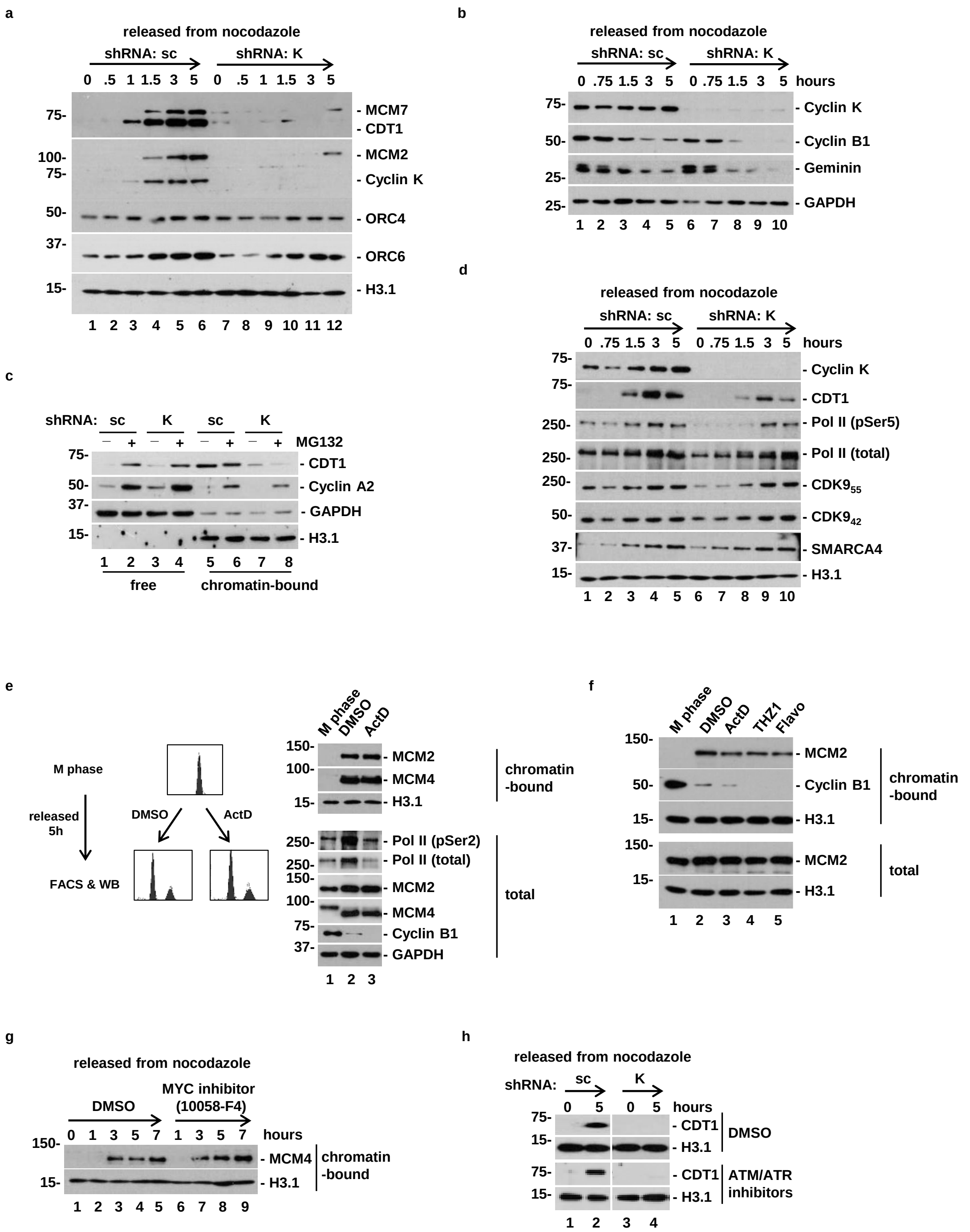

Supplementary Fig. 4: Dissection of potential mechanisms how cyclin K regulates pre-RC assembly.

(a) Analysis of the loading of ORC complex (ORC4 and ORC6) with or without cyclin K knockdown in HCT116 cells. Knockdown of cyclin K did not perturb the loading kinetics of ORC4 and ORC6. **(b)** Analysis of Geminin protein with or without cyclin K

knockdown in HCT116 cells. Knockdown of cyclin K did not perturb the kinetics of Geminin regulation in cell cycle. **(c)** Regulation of CDT1 by proteasome was similar with or without cyclin K knockdown in HCT116 cells. In addition, upregulation of CDT1 by MG132 treatment (5 μM) failed to rescue the effect by cyclin K knockdown. **(d)** Loading of several general transcription apparatuses was comparable with or without cyclin K knockdown in HCT116 cells, indicating that cyclin K knockdown did not cause a global defect in chromatin organization. **(e)** Inhibition of global RNA polymerase II transcription elongation by actinomycin D (ActD, 500 ng/ml) did not affect pre-RC assembly in HCT116 cells. **(f)** Inhibition of global RNA polymerase II transcription by different inhibitors did not affect pre-RC assembly in HCT116 cells. THZ1 (250 nM) inhibits CDK7 to block global transcription initiation, and reduces both Ser2 and Ser5 phosphorylation RNA polymerase II CTD. Flavopiridol (500 nM) inhibits CDK9 to block global transcription elongation and reduce Ser2 phosphorylation. **(g)** Inhibition of c-MYC (10058-F4, 50 μM) did not affect pre-RC assembly in HCT116 cells. **(h)** ATM and ATR inhibition failed to rescue CDT1 loading defect caused by cyclin K knockdown in HCT116 cells. Similar results were obtained from inhibition of ATM (KU-60019, 5 μM) and ATR (VE-821, 5 μM) in combination (shown here) or individually. All experiments were repeated at least three times and representative results are shown.

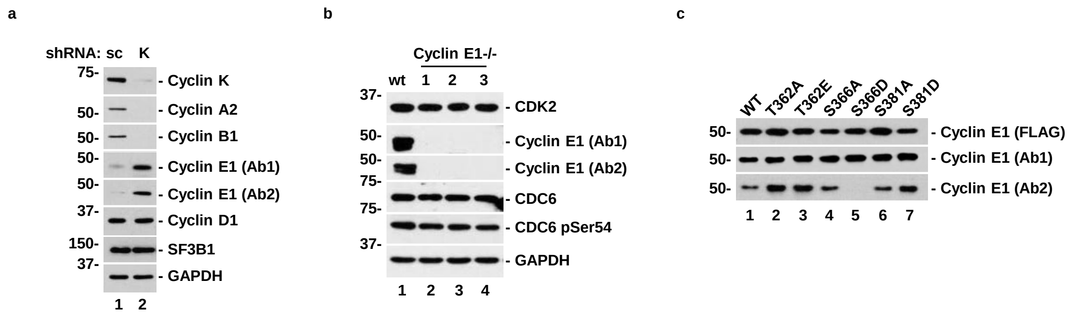

Supplementary Fig. 5: Characterization of various anti-cyclin E1 antibodies.

(a) Similar experiment as in Fig. 5a except that Ab1 was a different batch from the same commercial vendor. Note that the band above 50 kD denoted by asterisk in Fig. 5a and 5b was absent. **(b)** Verification of cyclin E1 antibodies using *cyclin E1* knockout HCT116 cell lines. **(c)** Total cell lysates were derived from HEK293 cells transfected with indicated cDNA, and analyzed by protein blotting with indicated antibodies. All constructs were FLAG-tagged at the C-terminus. WT, wild-type cyclin E1. All experiments were repeated at least three times and representative results are shown.

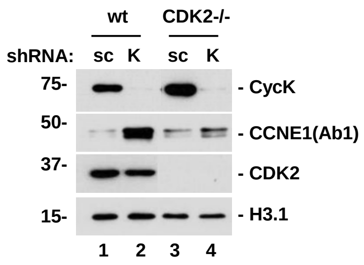

Supplementary Fig. 6: The accumulation of cyclin E1 induced by cyclin K knockdown is dependent on CDK2.

CDK2 knockout HCT116 cell lines (*CDK2*-/-) were generated by CRISPR/Cas9 technology. Total cell lysates were analyzed by immunoblotting with or without cyclin K knockdown for 3 days. wt, isogenic wild-type cells.

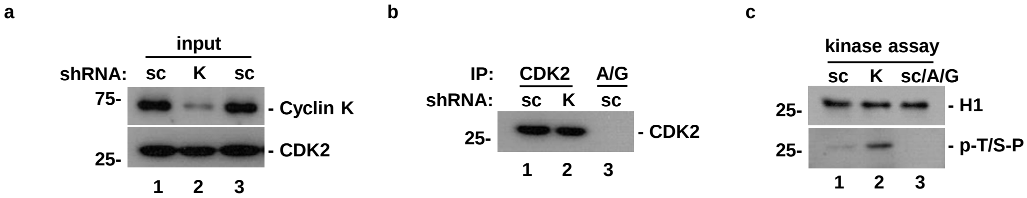

Supplementary Fig. 7: CDK2 kinase assay.

(a) Input of total cell lysates for anti-CDK2 immunoprecipitation generated from HCT116 cells with or without cyclin K knockdown. Cyclin K knockdown did not affect endogenous CDK2 protein level. **(b)** Endogenous CDK2 was immunoprecipitated from cell lysates shown in **(a)**, and normalized to equal amount for *in vitro* kinase assay. A/G, protein A/G agarose beads was used for mock immunoprecipitation without anti-CDK2 antibodies. **(c)** Recombinant human histone H1 (H1) was used as substrate for purified CDK2 from **(b)**. Phospho-T/S-P antibodies were used for detection of phosphorylated H1 as an indication of endogenous CDK2 kinase activity.

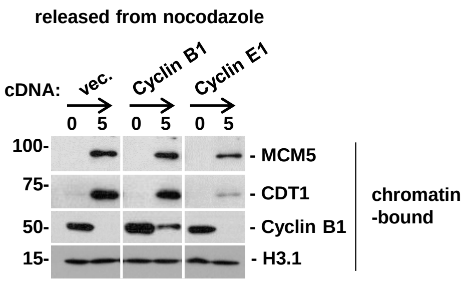

Supplementary Fig. 8: Ectopic expression of cyclin B1 does not compromise pre-RC assembly.

Either cyclin B1 or cyclin E1 was overexpressed in HCT116 cells, and pre-RC loading was similarly monitored as in Fig. 6a.

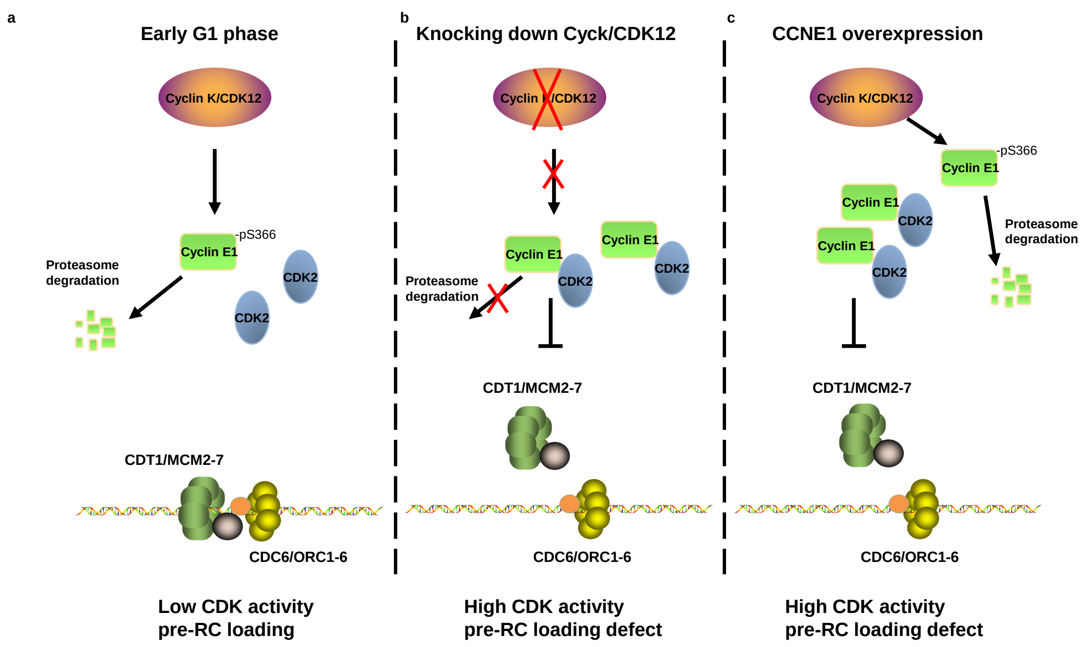

Supplementary Fig. 9: Working model how cyclin K regulates pre-RC assembly.

High level of CDK2/cyclin E1 activity in early G1 is detrimental to pre-RC assembly by preventing CDT1 and subsequent MCM loading. Under normal situation **(a)**, cyclin K/CDK12 directly or indirectly phosphorylates most cyclin E1 at S366 to prevent interaction with CDK2. Without interacting with CDK2, cyclin E1 is quickly degraded in cells so that CDK2 activity is low in G1, and pre-RC assembly is allowed. When cyclin K or CDK12 is knocked down **(b)**, S366 phosphorylation is lost, leading to increased cyclin E1/CDK2 activity in G1 to block pre-RC assembly. Likewise, when cyclin E1 is overexpressed **(c)**, there is not enough endogenous cyclin K/CDK12 to fully phosphorylates S366, leading to increased cyclin E1/CDK2 activity in G1. pre-RC assembly is compromised.

Supplementary Fig. 10: Full images of western blots.

Full unprocessed images and signals detected by western blotting, with the regions used in the corresponding main display items indicated by red frames.

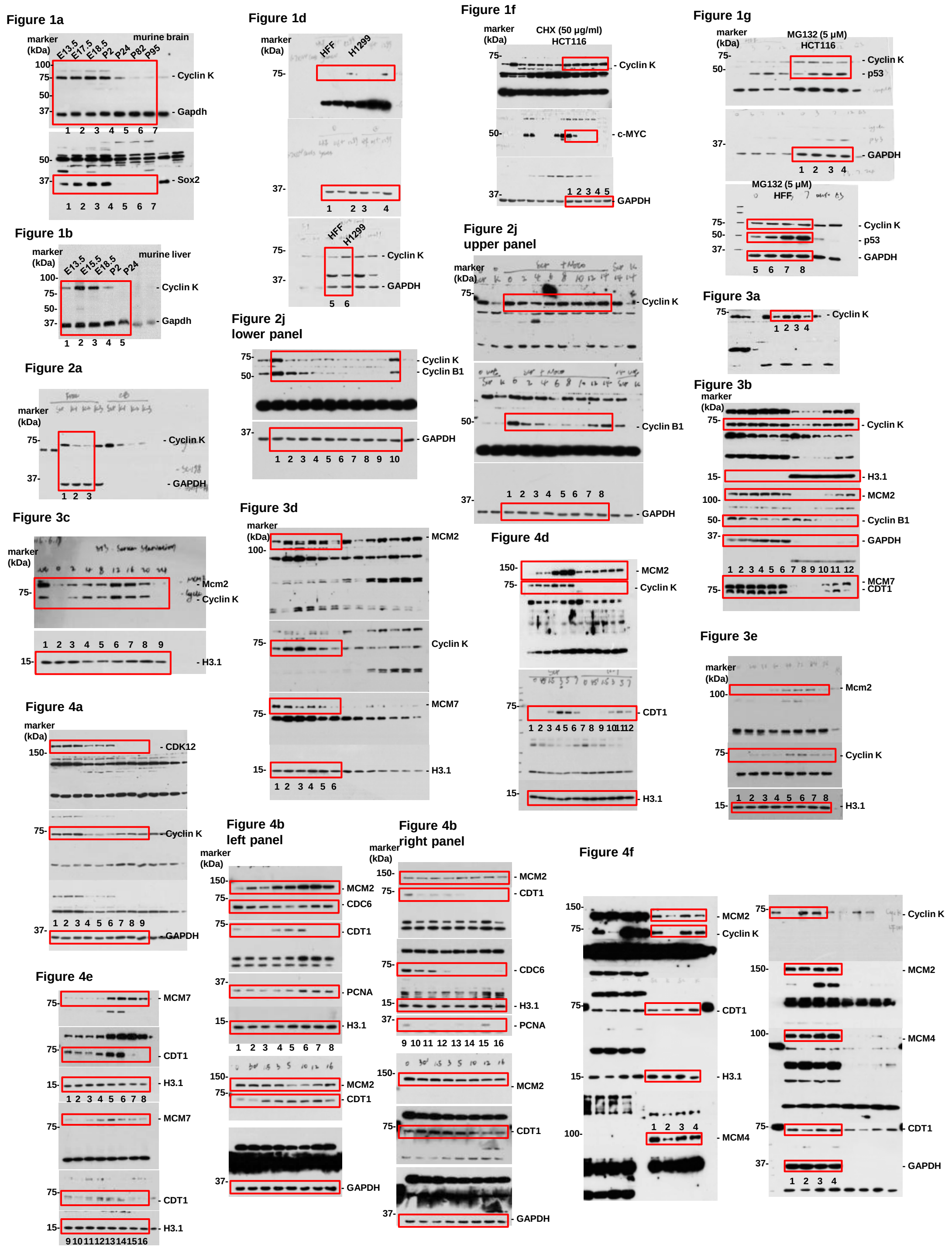

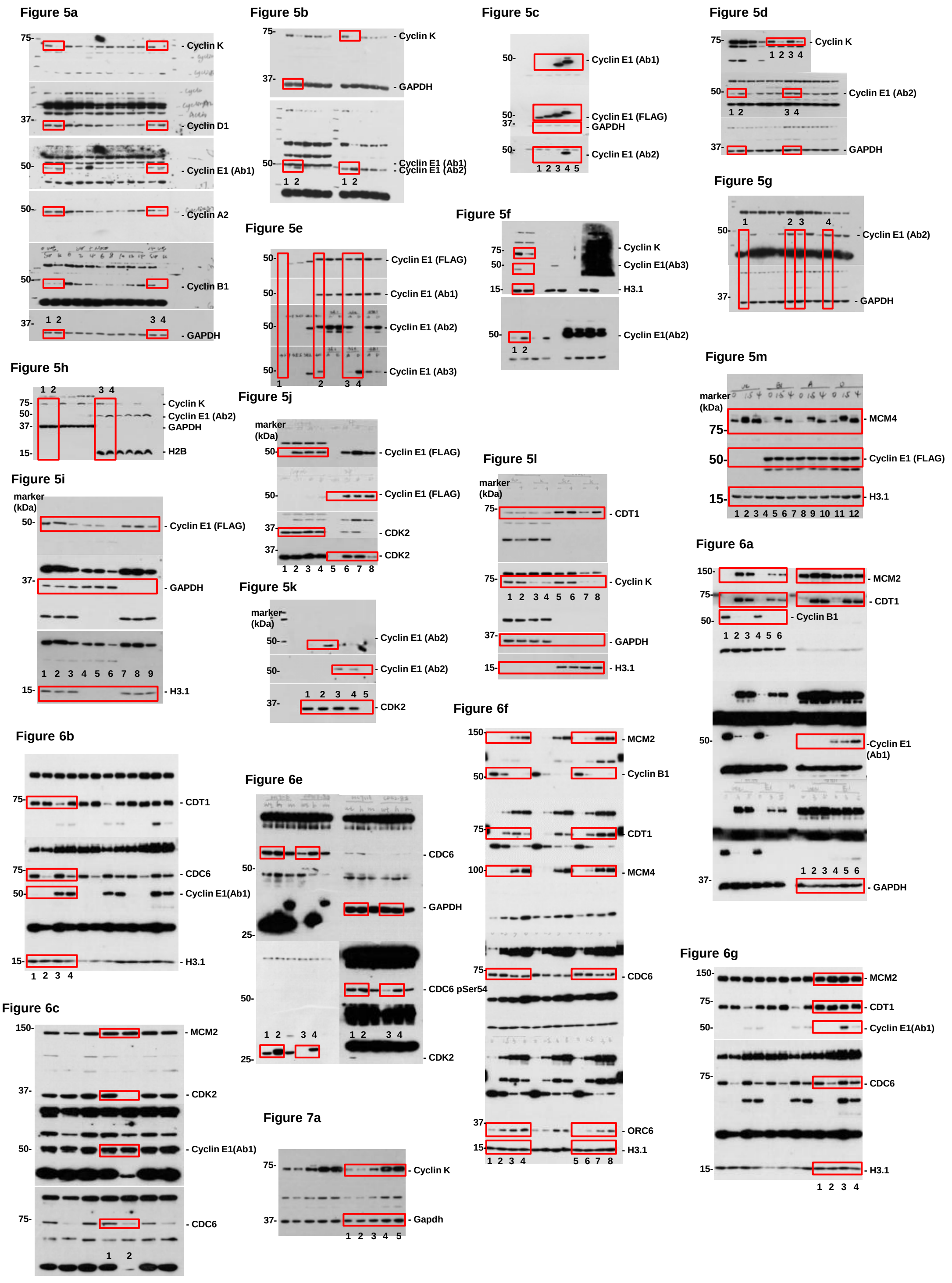
Figure 5a
Cyclin K
Cyclin D1
Cyclin E1 (Ab1)
Cyclin A2
Cyclin B1
GAPDH
Figure 5b
Cyclin K
GAPDH
Cyclin E1 (Ab1)
Cyclin E1 (Ab2)
Figure 5c
Cyclin E1 (Ab1)
Cyclin E1 (FLAG)
GAPDH
Cyclin E1 (Ab2)
Figure 5d
Cyclin K
Cyclin E1 (Ab2)
GAPDH
Figure 5g
Cyclin E1 (Ab2)
GAPDH
Figure 5e
Cyclin E1 (FLAG)
Cyclin E1 (Ab1)
Cyclin E1 (Ab2)
Cyclin E1 (Ab3)
Figure 5f
Cyclin K
Cyclin E1(Ab3)
H3.1
Cyclin E1(Ab2)
Figure 5h
Cyclin K
Cyclin E1 (Ab2)
GAPDH
H2B
Figure 5i
marker (kDa)
Cyclin E1 (FLAG)
GAPDH
H3.1
Figure 5j
marker (kDa)
Cyclin E1 (FLAG)
Cyclin E1 (FLAG)
CDK2
CDK2
Figure 5k
marker (kDa)
Cyclin E1 (Ab2)
Cyclin E1 (Ab2)
CDK2
Figure 5l
marker (kDa)
CDT1
Cyclin K
GAPDH
H3.1
Figure 5m
marker (kDa)
MCM4
Cyclin E1 (FLAG)
H3.1
Figure 6a
MCM2
CDT1
Cyclin B1
Cyclin E1 (Ab1)
GAPDH
Figure 6b
CDT1
CDC6
Cyclin E1(Ab1)
H3.1
Figure 6e
CDC6
GAPDH
CDC6 pSer54
CDK2
Figure 6f
MCM2
Cyclin B1
CDT1
MCM4
CDC6
ORC6
H3.1
Figure 6g
MCM2
CDT1
Cyclin E1(Ab1)
CDC6
H3.1
Figure 6c
MCM2
CDK2
Cyclin E1(Ab1)
CDC6
Figure 7a
Cyclin K
Gapdh

Suppl Fig 1a

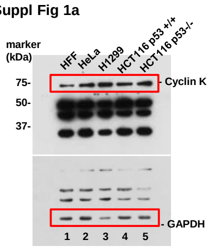

Suppl Fig 1b

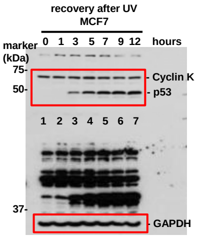

Suppl Fig 1c

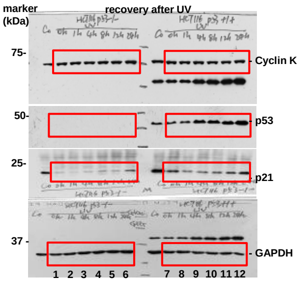

Suppl Fig 1d

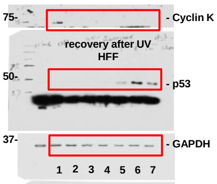

Suppl Fig 4a

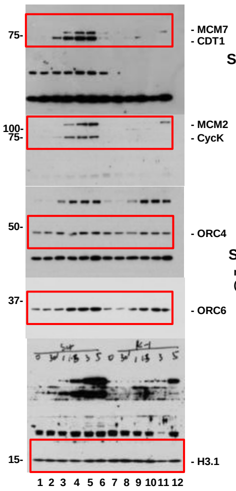

Suppl Fig 4b

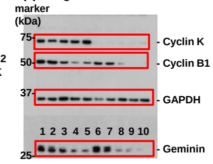

Suppl Fig 4c

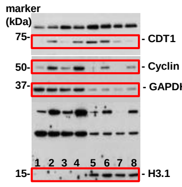

Suppl Fig 4d

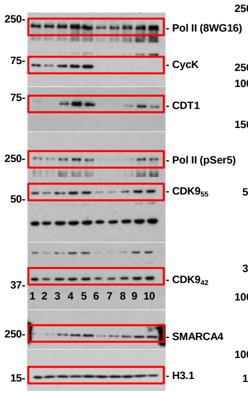

Suppl Fig 4e

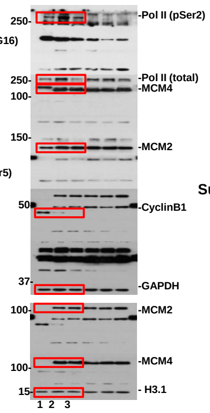

Suppl Fig 4g

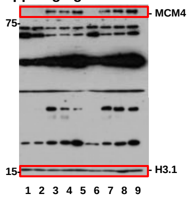

Suppl Fig 4h

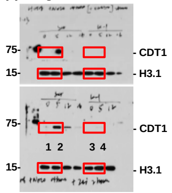

Suppl Fig 4f

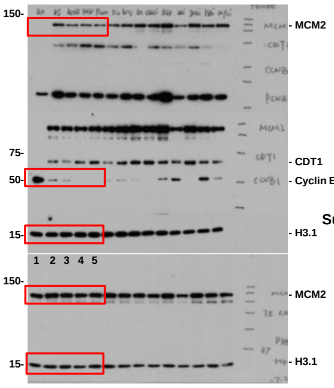

Suppl Fig 5a

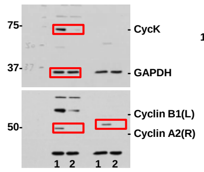

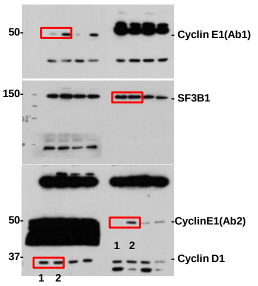

Suppl Fig 5b

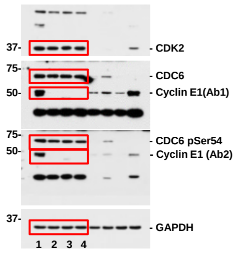

Suppl Fig 6

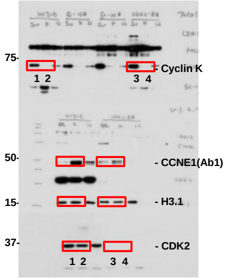

Suppl Fig 7

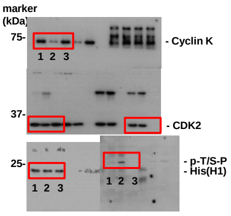

Suppl Fig 8

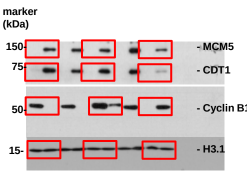

Suppl Fig 5c

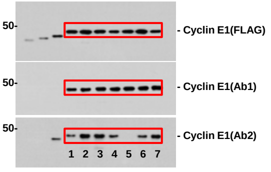

Supplementary Table 1: list of primes and oligos

Primers and oligos	Sequence 5'-3'
shRNA-Cyclin K targeting sequence 1	GCAGGACGTTTGTGCAAATTT
shRNA-Cyclin K targeting sequence 2	CCACCAAATCCTGGATCTTTA
shRNA-CDK12 targeting sequence	GCACTGAAAGAGGAGATTGTT
CCNE1 targeting gRNA1	CCTCGCCGTCCTGTCGATTT
CCNE1 targeting gRNA2	CCAAAATCGACAGGACGGCG
CDK2 targeting gRNA1	GGCGCTTAAGAAAATCCGCC
CDK2 targeting gRNA2	GATCTCTCGGATGGCAGTAC
PCR primers to validate Cyclin E1 KO	forward, ACACCATGAAGGAGGACGG<br>reverse, GCTGTGGCTGCATTAGAGA
PCR primers to validate CDK2 KO	forward, TATACTGCGTTCCATCCC<br>reverse, GCATTACCTTGATGAGGG
qPCR primers targeting CDS to validate Cyclin K knockdown	forward, AAAGACTTGGCTCATACACCC<br>reverse, CAGTTGCCAGGGTATCATAGTG
qPCR primers targeting 3' UTR to validate Cyclin K knockdown	forward, CAGCCTGGATGAGATAACGT<br>reverse, TTCCCATACTGCAACTGTCG
qPCR primers targeting CDS to validate CDK12 knockdown	forward, CTTGCTCGGCTCTATAACTCTG<br>reverse, TTCCCCAAGAATACATCCACAG
Cyclin K cloning	forward, GAGGATCCATGAAGGAGAATAAAGAAAAT<br>reverse, GATCTAGATTACTTGTCGTCATCGTCTTTG TAGTCTCTCATCCAGGCTGC
shRNA-resistant human Cyclin K	forward, CTCGCAGGACGTTTATGTAAGTTTGAAATACAAG<br>reverse, CTTGTATTTCAAACTTACATAAACGTCCTGCGAG

Cyclin B1 cloning	forward, GTTCTAGACACCATGGCGCTCCGAGTCACC<br>reverse, GTGGATCCTTACTTGTCGTCATCGTCTTTGTAGT<br>CCACCTTTGCCACAGCCTT
Cyclin E1 cloning	forward, GTGAATTCACCATGCCGAGGGAGCGCAG<br>reverse, GTTCTAGATTACTTGTCGTCATCGTCTTTGTAG<br>TCCGCCATTTCCGGCCCGCT
Cyclin E1 truncation (1-327) cloning	forward, GTGAATTCACCATGCCGAGGGAGCGCAG<br>reverse, GTTCTAGATTACTTGTCGTCATCGTCTTTGTAG<br>TCACAGTTCTCTATGTCGCA
Cyclin E1 truncation (1-355) cloning	forward, GTGAATTCACCATGCCGAGGGAGCGCAG<br>reverse, GTTCTAGATTACTTGTCGTCATCGTCTTTGTAGT<br>CTTCATCAGCGACGCCCCT
Cyclin E1 truncation (1-382) cloning	forward, GTGAATTCACCATGCCGAGGGAGCGCAG<br>reverse, GTTCTAGATTACTTGTCGTCATCGTCTTTGTAGTC<br>TTCAGACAACATGGCTTT
Cyclin E1 T362A mutation	forward, CACACAACATACAGGCCCACAGAGAC<br>reverse, GTCTCTGTGGGCCTGTATGTTGTGTG
Cyclin E1 T362E mutation	forward, CACACAACATACAGGAACACAGAGAC<br>reverse, GTCTCTGTGTTCCTGTATGTTGTGTG
Cyclin E1 S366A mutation	forward, GTCCAGCAAATCCAAGGCGTCTCTGTG<br>reverse, CACAGAGACGCCTTGGATTTGCTGGAC
Cyclin E1 S366D mutation	forward, GTCCAGCAAATCCAAGTCGTCTCTGTG<br>reverse, CACAGAGACGACTTGGATTTGCTGGAC
Cyclin E1 S381A mutation	forward, GAAAGCCATGTTGGCTGAACAAAATAGGG<br>reverse, CCCTATTTTGTTCAGCCAACATGGCTTTC
Cyclin E1 S381D mutation	forward, GAAAGCCATGTTGGATGAACAAAATAGGG<br>reverse, CCCTATTTTGTTCATCCAACATGGCTTTC
